# The Psychonauts’ World of Cognitive Enhancers

**DOI:** 10.3389/fpsyt.2020.546796

**Published:** 2020-09-11

**Authors:** Flavia Napoletano, Fabrizio Schifano, John Martin Corkery, Amira Guirguis, Davide Arillotta, Caroline Zangani, Alessandro Vento

**Affiliations:** ^1^ Department of Mental Health, Homerton University Hospital, East London Foundation Trust, London, United Kingdom; ^2^ Psychopharmacology, Drug Misuse, and Novel Psychoactive Substances Research Unit, School of Life and Medical Sciences, University of Hertfordshire, Hatfield, United Kingdom; ^3^ Swansea University Medical School, Institute of Life Sciences 2, Swansea University, Swansea, United Kingdom; ^4^ Psychiatry Unit, Department of Clinical and Experimental Medicine, University of Catania, Catania, Italy; ^5^ Department of Health Sciences, University of Milan, Milan, Italy; ^6^ Department of Mental Health, Addictions’ Observatory (ODDPSS), Rome, Italy; ^7^ Department of Mental Health, Guglielmo Marconi” University, Rome, Italy; ^8^ Department of Mental Health, ASL Roma 2, Rome, Italy

**Keywords:** cognitive enhancers, nootropics, novel psychoactive substances, novel psychoactive substances, screening, early warning systems

## Abstract

**Background:**

There is growing availability of novel psychoactive substances (NPS), including cognitive enhancers (CEs) which can be used in the treatment of certain mental health disorders. While treating cognitive deficit symptoms in neuropsychiatric or neurodegenerative disorders using CEs might have significant benefits for patients, the increasing recreational use of these substances by healthy individuals raises many clinical, medico-legal, and ethical issues. Moreover, it has become very challenging for clinicians to keep up-to-date with CEs currently available as comprehensive official lists do not exist.

**Methods:**

Using a web crawler (NPSfinder^®^), the present study aimed at assessing psychonaut fora/platforms to better understand the online situation regarding CEs. We compared NPSfinder^®^ entries with those from the European Monitoring Centre for Drugs and Drug Addiction (EMCDDA) and from the United Nations Office on Drugs and Crime (UNODC) NPS databases up to spring 2019. Any substance that was identified by NPSfinder^®^ was considered a CE if it was either described as having nootropic abilities by psychonauts or if it was listed among the known CEs by Froestl and colleagues.

**Results:**

A total of 142 unique CEs were identified by NPSfinder^®^. They were divided into 10 categories, including plants/herbs/products (29%), prescribed drugs (17%), image and performance enhancing drugs (IPEDs) (15%), psychostimulants (15%), miscellaneous (8%), Phenethylamines (6%), GABAergic drugs (5%), cannabimimetic (4%), tryptamines derivatives (0.5%), and piperazine derivatives (0.5%). A total of 105 chemically different substances were uniquely identified by NPSfinder^®^. Only one CE was uniquely identified by the EMCDDA; no CE was uniquely identified by the UNODC.

**Conclusions:**

These results show that NPSfinder^®^ is helpful as part of an Early Warning System, which could update clinicians with the growing numbers and types of nootropics in the increasingly difficult-to-follow internet world. Improving clinicians’ knowledge of NPS could promote more effective prevention and harm reduction measures in clinical settings.

## Introduction

Cognitive enhancement may be defined as “the amplification or extension of core capacities of the mind through improvement or augmentation of internal or external information processing systems” (1). Both non-pharmacological and pharmacological enhancers are sought by the general public in order to improve performance during studying and at work by increasing concentration, motivation and accuracy, *via* physical, behavioral and biochemical activities (2).

Cognitive enhancer drugs (CEs) are also known as “nootropics” (from the Greek ‘nous’ meaning ‘mind’ and ‘trepein’ meaning ‘turning/bending’), a term initially penned by Corneliu Giurgea when piracetam was found to exhibit memory-enhancing properties in clinical trials (3, 4). Cognitive enhancer drugs such as modafinil improve cognition in very specific ways such that it enhances “pattern recognition memory, digit span recall, and mental digit manipulation” (5).

### Cognitive Enhancers, Historical Perspective and State of the Art

Historically, CEs have been used to treat conditions related to cognition deficits such as Alzheimer’s disease, psychiatric disorders such as schizophrenia (6), stroke or attention deficit hyperactivity disorder (ADHD) (7–9). These phenomena commonly occur with aging (7–9). It was found that some CEs also improve cognitive functions in healthy subjects, such as memory, executive functions, creativity, and motivation (10). Their use has become more and more prevalent among college, high school, and university students as well as in the military (11–13).

The world of CEs is multifaceted and complex, with different molecules acting with different modes of actions and on different (and often multiple) receptors in the central nervous system (CNS). “Natural” enhancers such as nicotine (14–17) and caffeine (18) are generally accepted as substances that help us by improving focus, alertness, and productivity. Food-based antioxidants, herbal, and other food-derived nootropic agents have become increasingly popular in recent times after there have been suggestions of associations between cognition and diet (19). Prescription drugs, such as modafinil, amphetamine, and methylphenidate are used off-label by healthy people who do not have specific deficits but want to improve their standards of intellectual and cognitive performance (20). Cognitive enhancers also include many drugs which have never reached the market as they have been discontinued in Phase II or III clinical trials (7–9). The many dimensions of cognitive enhancement are described and disentangled in a recent review (2). Dresler and colleagues (2) pointed out how cognitive enhancement is not a monolithic phenomenon and how there are a great variety of interventions that can be classified and clustered into biochemical, physical, and behavioural enhancement strategies.

### Misuse of Cognitive Enhancers

The most prevalent CEs that are currently abused/misused include diverted prescription medicines such as those used for the treatment of attention deficit hyperactivity disorder (ADHD) *i.e.* methylphenidate (MPH) and amphetamine/dextroamphetamine (Adderall—most common brand); “wakefulness-promoting agents” with psychostimulant effects such as modafinil (21–23); illicit psychostimulants such as amphetamine, and drugs that act on the glutamatergic AMPA receptors, the so-called ampakines or “glutamate activators” (24). While the benefits of medications, such as MPH or modafinil, in patients suffering from specific diagnosed conditions (such as ADHD or narcolepsy) have been studied and evaluated, the potential benefits of these substances in heathy individuals remain unclear. The use of CEs in healthy individuals poses significant concerns due to the lack of clinical evidence regarding their safety, effectiveness, and social consequences, especially with long-term use.

Urban and Gao (24) emphasized that these newly misused drugs, *i.e.* MPH, may in fact improve cognition by acting on the memory and learning circuits, thus exciting the dopamine/glutamate/noradrenergic neurons. The modulation of these neurotransmitters in healthy individuals seeks to enhance their cognitive functions beyond baseline levels, but may also lead to paradoxical effects, particularly in children’s and adolescent’s growing brains (25). In these cases, glutamate modulation may impair behavior flexibility, which may facilitate addictive behaviors. Conversely, dopamine and norepinephrine reuptake inhibition may lead to a hyperdopamin-/hypernoradrenalin-ergic state, which may induce a cognition decline because the relationship between the prefrontal cortex cognition enhancement and the levels of both dopamine and noradrenaline is non-linear and actually an inverted U-curve (25–27). Urban et al. (28) have also emphasized that the use of CEs such as MPH and modafinil can have short- and long-term impacts on plasticity in the pre-frontal cortex that may affect the potential for plastic learning especially in children and adolescents.

Like many other NPS, nootropics have become increasingly easily available on the internet over the last 20 years. According to the United Nations Office on Drugs and Crime (UNODC) Early Warning Advisory (EWA) on new psychoactive substances (NPS), NPS have been reported from over 100 countries and territories from all regions of the world (29–32). In addition, the European Monitoring Centre for Drugs and Drug Addiction (EMCDDA) has been monitoring more than 700 NPS that have appeared on Europe’s drug market in the last 20 years, of which almost 90% have appeared in the last decade (33, 34). The European Database on New Drugs (EDND) of the EMCDDA records the notifications of new substances and the detection of NPS in Europe (35). Although many of these identified NPS might be used by healthy people as CEs, there are limited data on how many or which substance is, nor are CEs classified as a specific category. Despite being a challenging task in view of the pharmacological differences of CEs, producing a formal classification of these substances is crucial in order to further develop scientific research on the topic as well as regulate and monitor their use and effects.

### Previous Findings and Current Challenges

Scientific data regarding NPS used or misused as CEs are lacking. Recent research papers mostly focus on the misuse of specific and well-known CEs such as methylphenidate analogs (36, 37), designer benzodiazepines, phenmetrazine, modafinil, novel synthetic opioids (37), and MPH (38). More literature is available on CEs which are potentially able to address cognitive deficits in specific patient groups. Froestl and Maitre (39) have classified these molecules into 19 categories based on their pharmacodynamics. Some of these molecules could not be classified based on their pharmacodynamics and hence were classified based on their chemical structure or their origin *i.e.* as natural products or endogenous molecules (39). Many of these drugs were clinically tested for their potential to improve cognitive function. Although they all might have a potential for being misused by the general public looking to enhance their cognitive abilities, the vast majority of these molecules have never reached the market as most of them have been discontinued in Phase II or III clinical trials (7–9).

A comprehensive literature review completed by Froestl et al. (7–9) proposed a description and a classification of 1,705 molecules as “nootropic agents or CEs” in the Thomson Reuters Pharma database, which were studied for their potential to counter cognitive deficits in Alzheimer’s disease. The large number of CEs, reported in the latter review, is attributed to the fact that it contains a high proportion (42%) of molecules that were tested for the treatment of dementia and molecules which were discontinued. Many CEs were described as groups or families (*i.e.* beta-amyloid aggregation inhibitors). These CEs were not identified by either the EDND, EWA, or NPSfinder^®^ as this is not part of the remit of any of the NPS early identification systems. In particular, many categories of CEs described by Froestl et al. (7–9) such as “Drugs interacting with Cytokines”, “Drugs interacting with Gene Expression”, “Drugs interacting with Heat Shock Proteins”, “Drugs interacting with Hormones”, “Drugs interacting with Ion Channels (different from receptors)”, “Drugs interacting with Nerve Growth Factors”, “Drugs interacting with Transcription Factors”, “Metal Chelators”, “Drugs preventing amyloid-beta aggregation”, “Drugs preventing amyloid-beta aggregation”, “Drugs interacting with tau”, “Stem Cells” include molecules specifically targeted for Alzheimer’s disease and, therefore, less likely to be relevant for the NPS early identification systems.

Apart from the known families of CEs (historically derivatives of MPH, modafinil, and racetams), psychonauts (subjects who experience intentionally drug-induced altered states of consciousness (40) have been experimenting with a variety of commonly prescribed drugs as well as illicit substances, often finding subjective evidence of cognitive enhancement and sharing their knowledge within the dark web sites and surface internet fora. At present, a comprehensive, up-to-date list of currently available CEs does not exist. Moreover, CEs are not described as a specific category/family within the EDND or EWA databases; this is because many substances, with many different (complex and, sometimes, not fully understood) pharmacological mechanisms, have the potential of improving aspects of cognition. Finally, some of these substances are not illegal (*i.e.* prescribed medication, food supplements, natural remedies *etc.*). For these reasons, it is difficult to create an early identification system which is able to keep professionals up-to-date with the CEs which are currently available to the general public *via* the online market.

### Aims of the Study

In this study, the aims were to (a) identify and categorize the number of CEs collected by the NPSfinder^®^ web crawler from a range of psychonaut, NPS-related, online sources; (b) compare the NPSfinder^®^ cognitive enhancers’ list with related findings from the UNODC’s EWA and the EMCDDA’s EDND.

## Materials and Methods

### NPSfinder^®^, a Tool for the Early Recognition of NPS

NPSfinder^®^ is a crawling/navigating software which was designed to facilitate the early recognition of the continuously growing amount of NPS that are available on the internet. At present, NPSfinder^®^ is a password protected proprietary software, which allows registered researchers only to screen and classify the substances that are identified by the software. An open access part, which will allow the general public to have free access to the substances, is under development.

NPSfinder^®^ automatically scans the web for new/novel/emerging NPS, including CEs, *via* the identification of psychonauts’ websites/fora. Every time a new website is identified, all its items are scanned and compared with the online existing ones. When a novel substance is found, this is added to the growing NPSfinder^®^ database. NPSfinder^®^ screening process is tailored to each website, and no specific keywords are used by the software. This proprietary method, which was created by trained software engineers, allows to map, on a 24/7 basis, the large variety of psychoactive molecules mentioned/discussed within a range of representative online psychonauts’ web sites/fora. This list is continuously growing (the current, full list of these sites is available upon request).

NPSfinder^®^ was designed to extract a range of information regarding NPS, including: chemical and street names; chemical formulae; three-dimensional images and anecdotally reported clinical/psychoactive effects.

### Identification of Cognitive Enhancers by NPSfinder^®^

NPSfinder^®^ has been already successfully used to identify other types of NPS, including synthetic cathinones (41), novel psychedelics (42), and novel opioids (43). In each paper, the comparison with international or European NPS databases has shown that NPSfinder^®^ is able to identify substances which were not previously described by the existent early detection systems. Raising awareness of novel substances has important implications from both a legislative and a clinical perspective.

Between 26 November 2017 and 31 May 2019, NPSfinder^®^ carried out a range of open web crawling identification activities focusing on a large range of psychonaut-based, specialized, multilingual sources with a specific focus on new/traditional psychoactive substances of likely recreational interest. Although the language most typically used in these websites was English, further languages analyzed by NPSfinder^®^ included: Dutch, French, German, Italian, Russian, Spanish, Swedish, and Turkish. With the help of an *ad hoc* check control panel, all data were manually examined by four medically/psychiatrically-trained professionals (*i.e.* FN, DA, CZ, and LG). In this way, a full assessment and editing of each NPSfinder^®^ data item were conducted, and the range of unique CEs presented here was identified.

The collection of further information was completed by consulting a range of open libraries and chemistry databases referring to the index item, if existing. These data were then stored in an online, restricted access/password-controlled database located within firewall protected, highly secure, and consistently performing servers.

When any new item was detected during the automated web scan, the system sent an e-mail notification/alert to the core researchers’ mailing list. Data were then screened for relevance and possible duplications.

The identified psychoactive substances were classified as CEs when a cognitive enhancing ability of any kind (such as improved attention, concentration, alertness, and memory) was mentioned in the description and/or among the effects of the psychoactive substance. The used terms for the search were “nootropic”, “cognitive enhancers”, “cognition enhancement”, “smart drugs”, “memory enhancers”, “concentration enhancers”, “attention enhancers”, “neuro enhancers”, and “intelligence enhancers”. Therefore, it is to be noted that these identified CEs are thought by psychonauts as having cognitive enhancing properties according to their subjective and anecdotical experience rather than due to any pharmacological analysis.

When a substance that was identified by NPSfinder^®^ was not explicitly described as able to enhance cognitive abilities but was listed as a known CE within the comprehensive review by Froestl et al. (7–9), it was still included among the list of NPSfinder^®^ CEs.

### Identification and Classification of Cognitive Enhancers

The NPSfinder^®^ CE results (updated to May 2019) were compared with those reported by the UNODC’s EWA on NPS (updated by April 2019) and the EMCDDA’s EDND (updated by April 2019).

Using chemical structure identification and other published information (*i.e.* published research papers and official databases), researchers assigned each molecule to its drug class, using the classification described by Schifano et al. (44, 45) for NPS. This classification includes the following families: synthetic cannabimimetics, synthetic cathinones, novel psychostimulants, novel derivatives of classic psychedelics phenethylamines/MDMA-like drugs, synthetic opioids, synthetic cocaine substitutes, novel tryptamines derivatives, GABAergic drugs, phencyclidine-like dissociative drugs, piperazine derivatives, herbs/plants, prescribed drugs, and image and performance enhancing drugs (IPEDs*)*.

## Results

### Identification and Classification of CEs

After about 18 months of operation, the number of substances identified by the web crawler activities was 5,922. By the time of writing (January 2020), 4,204 unique NPS substances were included in the database, and 1,718 out of 5,922 (29.0%) remaining substances were found to be false positives or duplicates. The most common NPS mentioned in psychonauts’ fora included: psychedelic phenethylamines (30.1%); synthetic cannabimimetics (29.8%); and opioids (10.1%).

A total of 142 unique CEs was identified by NPSfinder^®^ (**Table A1**). Of these, 35 were explicitly described as having nootropic properties by psychonauts; the remaining 107 molecules were classified as CEs as also present in the comprehensive review on CEs written by Froestl et al. (7–9).

Using the classification described by Schifano and colleagues (44, 45), the CEs identified by NPSfinder^®^ (n = 142) were divided into 10 categories; the majority of these substances were classified as plants/herbs/products (29%), prescribed drugs (17%), image and performance enhancing drugs (IPEDs) (15%), and psychostimulants (15%); in addition, there were substances classified as miscellaneous (8%), phenethylamines (6%), GABAergic drugs (5%), cannabimimetic (4%), tryptamines derivatives (0.5%), and piperazine derivatives (0.5%) (**Table 1**).

**Table 1:** CEs identified by NPSfinder^®^ using Schifano et al.’s (44) classification (n=142).

Class (44)	N. of CEs
Plants/herbs/products	41 (29%)
Prescribed drugs	24 (17%)
Image and performance enhancing drugs (IPEDs)	21 (15%)
Psychostimulant drugs	21 (15%)
Miscellaneous	11 (8%)
Phenethylamines	9 (6%)
GABAergic drugs	7 (5%)
Cannabimimetic	6 (4%)
Tryptamines derivatives	1 (0.5%)
Piperazine derivatives	1 (0.5%)
**TOTAL**	**142**

### Comparison of NPSfinder^®^ Findings With EU and UN NPS-Related Databases

Current NPSfinder^®^ results were compared with the EMCDDA and the UNODC databases in order to ascertain which molecules were also detected and listed by the official European and United Nation early identification systems.

Out of the 142 molecules identified as CEs by NPSfinder^®^, a total of 105 chemically different substances were uniquely identified by NPSfinder^®^; of the remaining 37 molecules, 22 were also listed in both the EDND and EWA databases, 15 of which were reported in both the NPSfinder^®^ and in either the EMCDDA (n = 11) or the UN databases (n = 4) (**Table A1**).

Only one CE was uniquely identified by the EDND (*MIQ-001, also called meta-IQ*); no CE was uniquely identified by the EWA database.

### CEs Identified According to Their Identification Source

**Figure 1:**
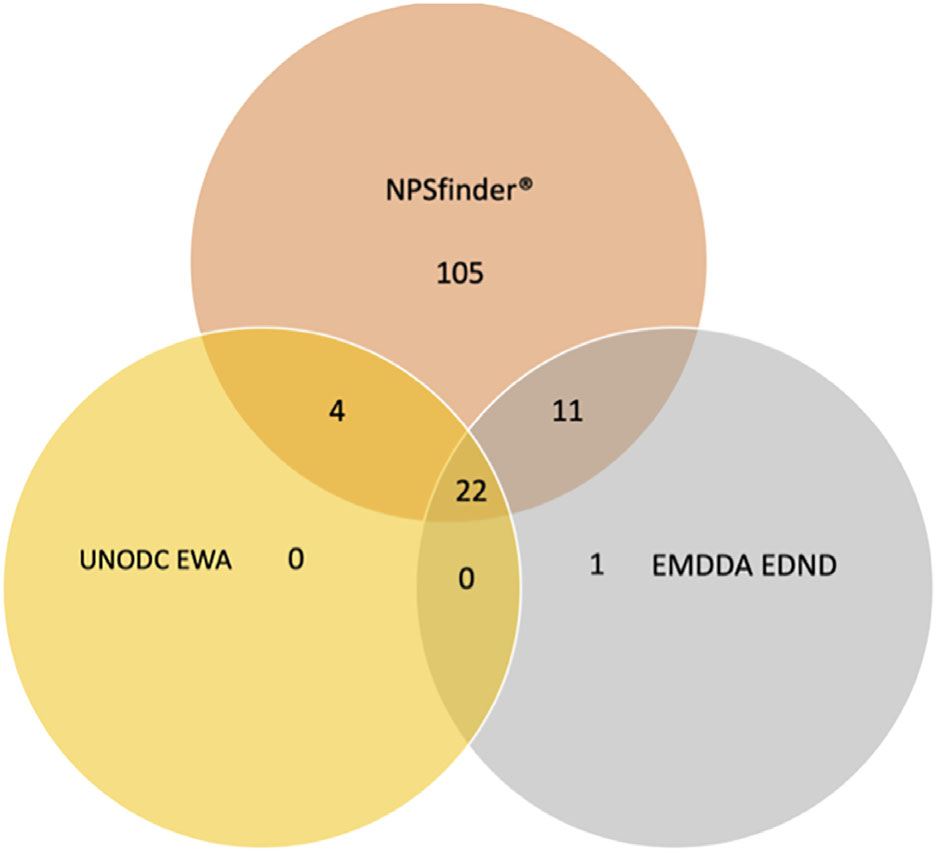
Number of CEs identified by EMCDDA, UNODC, and NPSfinder^®^ according to their identification source (n = 1,785).

**Figure 1** shows the number of CEs identified by each source (including NPSfinder^®^, EDND and EWA database) as well as the ones identified by more than one source. A full list of the CEs is available upon request.

## Discussion

In this paper, we aimed to evaluate whether the innovative crawling software NPSfinder^®^ can be employed as a helpful tool in the early identification and prediction of CEs. In order to achieve this goal, findings from NPSfinder^®^ were cross-checked with two official sources (EMCDDA’s EDND and UNODC’s EWA). To the best of our knowledge, this is an unprecedented list of drugs which are described as CEs and, therefore, with a potential for recreational misuse by healthy individuals.

NPSfinder^®^ identified 35 molecules (out of the total of 4,204) that were described by psychonauts as having cognitive enhancing effects, such as improved memory, alertness, attention, and concentration. A further 107 molecules were previously described as CE (7–9), although psychonauts did not explicitly describe them as CE. Since psychonauts experiment with novel substances in order to intentionally experience altered states of consciousness, it is to be expected that their interest also extends to the world of CEs. Among the CEs that they have been discussing online, there are mostly molecules that are known to have nootropic properties, are not illegal, and are likely to be easily available on the market (such as racetam compounds, modafinil and its derivatives, methylphenidate and its derivatives and food supplements). Our results showed that NPSfinder^®^ could be employed as an Early Warning System tool to help clinicians with keeping their knowledge up-to-date with the growing numbers and types of nootropics in the increasingly difficult-to-follow online market.

It is not surprising that the included sources (*i.e.* NPSfinder^®^, EDND, and EWA) have identified mis-matching numbers and types of CEs, as they differ in their methodology and purposes of CE identification. In fact, the EDND was created in order to allow the European Union to rapidly detect, assess, and respond to health and social threats caused by NPS (35). The UNODC EWA on NPS provides access to basic information on new psychoactive substances, including trend data, chemical details on individual substances, supporting documentation on laboratory analysis and legislative responses (30). Specifically, the EDND and EWA focus on illegal drugs and do not look at websites that contain patented medications, while NPSfinder^®^ looks at websites whose contributors might have accessed sources containing patent medications.

### NPSfinder^®^ Findings

The large number of molecules that are both identified by NPSfinder^®^ and described by Froestl et al. (7–9) leads us to believe that nowadays psychonauts are discussing (and likely using) substances that have been considered or used for the treatment of the Alzheimer’s disease over seven years ago, and they are doing so in order to improve their cognitive performances in the absence of clinical reasons.

Among the CEs that have been subjectively identified by psychonauts as able to improve certain aspects of their cognition, there are molecules whose objective cognitive enhancing properties have not been established by research studies, such as the selective serotonin re-uptake inhibitors (SSRIs), melatonin and many others.

### Comparison of NPSfinder^®^ Findings With EDND or EWA Databases

The large number of unique molecules that were uniquely identified by NPSfinder^®^ can be explained with the innovative methodology that NPSfinder^®^ used for the early identification of all NPS, including CEs (41–43). Being a dynamic software, NPSfinder^®^ is able to automatically scan the web for new/novel/emerging NPS on a 24/7 basis. This is indeed an effective mechanism for the early identification of (potential) NPS, which are being discussed on the psychonauts’ websites and fora.

### Description and Classification of CEs Identified by NPSfinder^®^

The CEs identified by NPSfinder^®^ (n = 142) were divided into 10 categories as shown in **Table 1**.

#### Plants/herbs/product:

The NPSfinder^®^ family of “Plants/herbs/product” contains a list of plant-based substances with a variety of psychoactive ingredients (**Table 2**).

**Table 2:** Plants/herbs/product (n=41; 29%).

1	Acetyl-L-carnitine
2	Areca nut
3	Arecoline (transdermal patch)
4	Bacopa monnieri
5	Caffeine
6	Catechins
7	Celastrus paniculatus
8	Cinnamon extract
9	Coumarins
10	Curcumin
11	Flavonoids
12	Ginger root extracts
13	Gingko biloba
14	Ginseng
15	Harmaline
16	Harmalol
17	Icariin
18	Kaempferol
19	Kava kava
20	Kratom
21	Lemon balm
22	Lobeline
23	Maca
24	Marijuana
25	Menthol
26	Mucuna pruriens
27	Naringin
28	Nicotine (patch)
29	Peganum harmala
30	Periwinkle
31	Quercetin
32	R-alpha-lipoic acid
33	Sakae naa
34	St John’s wort
35	Tannic acid
36	Vitamin A
37	Vitamin B12
38	Vitamin D
39	Withania somnifera
40	Yerba Mate
41	Yohimbine HCL

In this group, there are many well-known substances such as: caffeine, nicotine, cinnamon, ginger root extracts, curcumin, ginseng, coumarins, menthol, St John’s wort, Yerba mate, Bacopa monnieri, Areca nut (and its main active ingredient arecoline), Lemon balm, Mucuna pruriens, Peganum harmala, harmaline, harmalol, and lobeline, some of which are commonly used by ayurvedic traditional medicine or in other branches of alternative medicine to improve memory and/or to treat various diseases. Flavonoids such as quercetin and naringin, as well as vitamins A, B, and D are also part of this group.

There are studies on the cognitive enhancing properties of *caffeine* (18), *nicotine* (14–17), *curcumin* (46–48), *St John’s wort* (*Hypericum perforatum*) (49), *Bacopa monnieri* (50), and many others. Perry and Howes (51) completed an informative review on medicinal plants in dementia, pointing out the potential cognitive benefits of a significant variety of plants and herbs. A recent systematic review has found that *tyrosine* and *caffeine* could enhance cognitive performance when healthy young adults are sleep-deprived in a military context (52).

#### Prescribed drugs:

*Methylphenidate* is undoubtedly the most prescribed CE, and being indicated for the treatment of ADHD in many countries, it is described, in this paper, within the “prescribed drugs” group. The non-medical use of methylphenidate as a CE, which involves an attempt to improve memory, increase mental concentration, control anxiety, and stimulate motivation and creativity, is rising worldwide (38, 53). Many other prescribed drugs are being talked about in psychonauts’ blogs and fora (**Table 3**).

**Table 3:** Prescribed drugs (n=24; 17%).

1	123I-Ioflupane
2	Amphetamine/dextroamphetamine (Adderal)
3	Armodafinil
4	Atomoxetine
5	Dextroamphetamine
6	DL-Phenylalanine
7	Galantamine
8	Hydergine
9	Lisdexamfetamine
10	Melatonin
11	Memantine
12	Memantine extended release
13	Methylphenidate
14	Modafinil
15	Modafinil suphone
16	NSI-189
17	Quetiapine
18	S-adenosyl-methionine
19	Selegiline
20	Sildenafil
21	Stablon
22	Tadalafil
23	Tropicamide
24	Vasopressin

Among the “prescribed drugs” family described by NPSfinder^®^ the SSRIs are also listed as a class. Research studies have often failed to demonstrate that SSRIs can have cognitive enhancing properties (54, 55). For example, neither *sertraline* (54) nor *citalopram* (55) appeared to be superior to placebo in improving cognition in patients with Alzheimer’s disease and comorbid depression. It was also suggested that any cognitive benefits of SSRIs were likely to be secondary to their effect on mood or behavioral disturbances. However, a more recent review on the topic concluded that the lack of evidence for SSRIs as CEs or disease modifiers in Alzheimer’s disease is more the result of omissions in clinical trial design, as opposed to reports of negative evidence (56). Interestingly, both *fluoxetine* and *methylphenidate* potentiate gene regulation in the striatum, and their combination seems to mimic cocaine effects, with related increased risk for substance use disorder (57).

It is possible that many prescribed drugs are currently being misused by the general public but not picked up by the regulatory bodies because the vast majority of them are not classified as illegal. It is important that more studies and cross-sectional surveys are conducted as well as that the current pharmacovigilance systems focus on determining current patterns and quantifying current usage of these drugs by healthy people.

#### Image and Performance Enhancing Drugs (IPEDs):

Racetam compounds, which are classically one of the major CE family (58), are identified by NPSfinder^®^ and listed within the IPEDs sub-group (**Table 4**).

**Table 4:** IPEDs (n=21; 15%).

1	Acetildenafil
2	Alpha GPC
3	Aminotadalafil
4	Aniracetam
5	Centrophenoxine
6	Choline Bitartrate
7	Citicoline
8	Creatine
9	Coluracetam
10	Dehydroepiandrosterone
11	Fasoracetam
12	Homosildenafil
13	Huperzine A
14	Lovegra
15	Nefiracetam
16	Noopept
17	Oxiracetam
18	Phenylpiracetam
19	Piracetam
20	Pramiracetam
21	Vardenafil

*Piracetam* enhances cognitive function without causing sedation or stimulation (3). This drug is also being used in clinical practice for the treatment of several diseases (59–62) although its mechanism of action remains not fully understood.

NPSfinder^®^ identified *aniracetam, coluracetam, fasoracetam, nefiracetam, oxiracetam, phenylpiracetam, piracetam, and pramiracetam*. Although all these substances have been mentioned in the psychonauts’ fora as having nootropic properties, research studies have not always succeeded in demonstrating their cognitive enhancing qualities. For example, recent studies failed in showing that *aniracetam* improves working memory in pigeons (63), learning and memory in rats (64), or cognitive and affective behavior in mice (65). Moreover, *nefiracetam* did not prove to be more efficacious than placebo in ameliorating apathy in stroke (66) despite some positive pre-clinical results (67, 68). One old study on *pramiracetam* has failed to demonstrate any cognitive benefit from its administration to patients suffering from Alzheimer’s disease (69). There are no available studies on *coluracetam, fasoracetam*, and *phenylpiracetam.*

#### Psychostimulant drugs:

Among the psychostimulant CEs are described many derivatives of *methylphenidate* and *modafinil* (**Table 5**). These have been listed in this group when not licensed as prescribed drugs.

**Table 5:** Psychostimulants drugs (n=21; 15%).

1	3,4-Dichloromethylphenidate
2	4 CTMP
3	4-fluoromethylphenidate
4	4-Mthylmethylphenidate
5	Adrafinil
6	Benzyl cyanide
7	Dexmethylphenidate
8	Dimethylaminoethanol
9	Ethylphenidate
10	Fladrafinil
11	Flmodafinil
12	L-655,708
13	Methylmorphenate
14	Methylnaphthidate
15	N-Methyl-4,4’-Difluoro-Modafinil
16	N-Methyl-cyclazodone
17	Pemoline
18	Prolintane
19	Razobazam
20	RO-4938581
21	Tyrosine

*Methylphenidate* is a prescription drug with medical restrictions in several countries, therefore, many illegal analogues have emerged on the internet and darknet drug markets during the last few years (53). The derivatives of methylphenidate that have been identified by NPSfinder^®^ include: *3,4-dichloromethylphenidate, 4-fluoromethylphenidate, 4-methylmethylphenidate, dexmethylphenidate, ethylphenidate, methylmorphenate*, and *methylnaphthidate.*

The derivatives of modafinil include: adrafinil, fladrafinil, flmodafinil, and N-methyl-4,4′-difluoro-modafinil.

#### Miscellaneous:

The categories “miscellaneous” include amino-acids such as *tryptophan and L-tryptophan, 5-hydroxytryptophan, phenylalanine*, and *theanine*, as well as man-made chemicals such as *vinpocetine* and *sulbutiamine* and other various molecules such as *beta-asarone, PRE-084*, and *RO-4491533*. No research studies are available regarding the misuse of these molecules by healthy subjects in order to ameliorate their cognitive function (**Table 6**).

**Table 6:** Miscellaneous (n= 11; 8%).

1	5-HTP
2	Beta-asarone
3	L-Tryptophan
4	Phenylalanine
5	PRL-8-53
6	PRE-084
7	RO-4491533
8	Sulbutiamine
9	Theanine
10	TRP
11	Vinpocetine

#### Phenethylamines:

The phenethylamines-related compounds that have been identified by NPSfinder^®^ are listed in **Table 7**.

**Table 7:** Phenethylamines (n=9; 6%).

1	2C-D
2	B-HO-Hordenine
3	Desoxypipradrol
4	Ephedrine
5	Geranamine
6	Hordenine
7	Isopropylphenidate
8	Octopamine
9	Propylphenidate

These are stimulant, entactogenic, and hallucinogenic substances that share similar chemical structures with amphetamine, catecholamines, synthetic cathinones, and other molecules (70).

Phenethylamines are known to enhance mood and empathy in healthy subjects. Substituted phenethylamines also include substituted amphetamines, which have been used as CEs to promote learning and memory but can ultimately lead to addiction (20). Dolder et al. (20) found that MDMA-induced subjective, emotional, sexual, and endocrine effects that were clearly distinct from those of *methylphenidate* and *modafinil*. To the best of our knowledge, there are no research studies or case reports focusing on the misuse of specific phenethylamines as CEs by healthy subjects.

#### GABAergic drugs:

GABAergic drugs are chemicals that produce their effects *via* interactions with the GABA system, such as by stimulating or blocking neurotransmission (71).

Among these, *GABA*, *tolibut*, *picamilon*, *phenibut*, and *f-phenibut* were discussed in the psychonauts’ fora as having tranquillizing as well as nootropic properties (**Table 8**). There is increasing evidence suggesting that *phenibut*, a potent psychoactive substance with GABA-B agonist properties which is often sold as a “dietary supplement”, can induce withdrawal and physical dependence which makes its use dangerous (72–76). *f-phenibut*, which is closely related to *phenibut*, is a central nervous system depressant (72); *tolibut* is a GABA analog that was developed in Russia (77), similarly to *picamilon*, which is formed by a synthetic combination of niacin and *γ*-aminobutyric acid (GABA). *Picamilon* was developed in the Soviet Union in 1969 (78) and further studied in both Russia (79) and Japan (80) as a prodrug of GABA.

**Table 8:** GABAergic drugs (n=7; 5%).

1	F-Phenibut
2	GABA
3	Phenibut
4	Picamilon
5	PWZ-029
6	SH-053-R-CH3-2′F
7	Tolibut

#### Cannabimimetic:

Among Cannabimimetic drugs there are the synthetic cannabimimetics that are designer drugs that target the same receptors to which cannabinoids in cannabis plants, tetrahydrocannabinol (THC) and cannabidiol (CBD) bind (81, 82). *dexanabinol*, *drinabant*, *Dronabinol*, *JZL-184*, *rimonabant*, and *URB-597* were the six CEs belonging to this group that were identified by the NPSfinder^®^ (**Table 9**).

**Table 9:** Cannabimimetic (n=6; 4%).

1	Dexanabinol
2	Drinabant
3	Dronabinol
4	JZL-184
5	Rimonabant
6	URB-597

The use of cannabimimetics as CEs seems counter-intuitive as both pre-clinical and human studies have found a link between consumption of cannabinoids and long-term deficits of cognitive functions, especially high-order cognitive functions (83–88). However, recent pre-clinical studies have found that delta-9-THC can improve cognitive performances in rats (89) and mice (90). THC, in fact, appears to promote hippocampal neurogenesis to prevent neurodegenerative processes occurring in animal models of Alzheimer’s disease, to protect from inflammation-induced cognitive damage, and to restore memory and cognitive function in old mice (91).

#### Tryptamines derivatives:

*5-Methoxytryptamine* (*5-MT*, also called *mexamine*) (**Table 10**) was the only tryptamine derivative identified by NPSfinder^®^ (as well as by the EWA). This is a tryptamine derivative closely related to both the serotonin and melatonin neurotransmitters (92). To the best of our knowledge, there are no studies, surveys, or case reports that identified 5-MT as a drug used by healthy people in order to improve their cognitive abilities. Jansen et al. (93) reviewed the efficacy of melatonin in addressing cognitive impairment in dementia but found the evidence for this to be inconclusive.

**Table 10:** Piperazine derivatives (n=1; 0.5%).

1	Fipexide

#### Piperazine derivatives:

*Fipexide* (also known as *attentil* and *vigilor*) (**Table 11**) is the only substitute piperazine that has been identified by NPSfinder^®^ as a CE. This was initially developed in Italy in 1983 (94) and used as a CE in Italy and France for the treatment of dementia (95). *Fipexide* is no longer in use due to the occurrence of rare side-effects (96, 97). On psychonauts’ fora it is described as a molecule able to improve short term memory, attention, learning, and cognition.

**Table 11:** Tryptamine derivatives (n=1; 0.5%).

1	Mexamin

### Ethical, Clinical and Legal Issues

Ethical issues raised by cognitive enhancement have been debated for over a decade (98), and many experts have identified multiple ethical concerns including risks to mental and health safety (99). While CEs hold significant benefits in improving cognitive impairments in several neuropsychiatric disorders such as Alzheimer’s disease (7–9) and schizophrenia (100), the use of nootropics by healthy individuals clearly poses ethical, clinical, and legal issues, as well as the need to develop a practical policy framework.

Mohamed and Sahakian (101) pointed out that CEs’ use in healthy people might have some advantages, such as: helping reduce disparity in society by mitigating the adverse environmental effects (like poverty) on the brain; improving the performances of people who need to perform at the best of their abilities in every situation (such as surgeons or pilots); finally CEs might also be used by people with undiagnosed disorders (such as ADHD) who might be therefore self-medicating with stimulant medications.

On the other hand, it is of concern that the safety and efficacy of these drugs in healthy individuals in the long-term are still unclear. While some CEs have been studied and research data on their mechanism of action and potential benefit are available, the action, the beneficial effects, and the potential side-effects of the majority of them have yet to be fully described and understood. Furthermore, CEs’ effects (if present at all) seem to be temporary, lasting until their metabolism and elimination (102). Some of these drugs can cause dependence and have a significant range of harmful effects; they can be particularly dangerous to young people as their brains are not fully developed. Studies producing null results and some evidence of task-specific impairments should be also noted (103).

The limited evidence of effectiveness as well as the potential side-effects should be cautiously considered by relevant legislative and regulatory bodies. In 2015, the US Presidential Commission for the Study of Bioethical Issues (104) released a report on CE, reporting up-to-date findings and providing recommendations for clinicians (104). The Australian Alcohol and Drug Foundation has recently raised doubts about the actual cognitive benefits of most CEs, indicating that scientific studies showed only little to no benefits for cognitive enhancement in healthy individuals, while the associated side-effects do pose significant risks to health and safety of the general public (105).

While further research is needed on the topic, the early identification of CEs that are most commonly discussed on the internet will increase clinicians’ awareness of this phenomenon and potentially help them make clinical decisions for patients presenting with psychiatric symptoms or physical health problems related to these substances. NPSfinder^®^ could also be an important tool for analytical toxicologists to focus their efforts on the detection of the most recently misused substances (106, 107).

## Limitations

In the online world, a significant variety of molecules/substances are described as CEs by anecdotal report or unofficial sources; it is important to note that the list of CEs is constantly evolving and changing. An official, up-to-date, comprehensive list of CEs is not currently available in the literature. The Early Warning Systems fail in the early detection of these substances as they are mostly legal products such as food supplements or prescribed medication, which are misused by healthy individuals to improve their cognitive abilities.

In addition, there is a lack of an official classification of CEs in families/categories. We based our classification on the one described by Schifano et al. (44). We noted that another type of classification, such as the one described by Froestl et al. (7–9) which is based on substances’ pharmacodynamics properties, is also relevant and useful and could be used when further data on NPS pharmacological properties will be available.

In fact, many CEs do not have a fully understood mechanism of action, which makes it difficult to link them to a specific category; other CEs have multiple mechanisms of actions (*i.e.* might target several different receptors), and they could therefore belong to more than one category; for example, one CE might belong both to the “prescription drugs” and the “GABAergic drugs” groups. Some of the categories can themselves be very broad and have different types of molecules belonging to it, for example “IPEDs”.

Furthermore, it is important to note that a limited number of languages were used for the screening of molecules on the web, and although many substances were first identified in seizures in Asia, only European languages are used. For all these reasons, forming a comprehensive and definite classification of CEs remains a complex challenge.

## Conclusions

In this paper, three different databases, including the innovative crawling software (NPSfinder^®^) and two official sources (EMCDDA’s EDND, UNODC’s EWA) were cross-checked.

CEs are a wide and diverse group of molecules, constantly growing in terms of numbers as well as availability among the general public and especially *via* online platforms. CEs differ for pharmacological activity, time, and mode of action, targeted cognitive domain, pharmacodynamic and pharmacokinetic properties, as well as possible short- and long-term side-effects. The popularity of chemicals that are potentially able to augment brain functions is not surprising in a society which constantly demands for increasingly high cognitive performances.

For the current official Early Warning Systems, it is challenging to identify and monitor the use of CEs as they are often sold as legal food supplements or as prescribed medication for a number of medical conditions. Due to its innovative methodology, NPSfinder^®^ has demonstrated its ability to identify a higher number of CEs than the official EMCDDA’s EDND and UNODC’s EWA (108). For this reason, NPSfinder^®^ can be considered as a helpful systematic tool which could update clinicians with the growing numbers and types of nootropics in the increasingly difficult-to-follow internet world.

Previously, Arillotta and colleagues (43) have identified 176 novel opioids which were not listed in either international or European NPS databases, such as EMCDDA or UNODC. This information is useful to stakeholders such as enforcement agents, emergency department, scientific community, prevention program setters, and other regulatory agencies. The same applies to CEs; in particular, the early identification of substances that are misused as CEs and the discovery of novel CEs that were never reported or identified before are crucial to raise the awareness of regulatory bodies. The identification of a drug is key to the treatment of its potential physical and psychiatric effects; if the drug is novel, its description may shed some light on its pharmacokinetics and toxicodynamics, which would in turn inform treatment decision-making in clinical settings.

Improving clinicians’ knowledge of CEs is of paramount importance, and further research in order to clarify mechanism of actions, as well as short- and long-term effects of many CEs is also needed. The early identification and better understanding of the distribution and effects of CEs could promote both more effective prevention and harm reduction measures in clinical settings, including emergency departments, mental health and general practice clinics.

## Data Availability Statement

The raw data supporting the conclusions of this article will be made available by the authors, without undue reservation.

## Ethics Statement

The current study involving human participants were reviewed and approved by the University of Hertfordshire Ethics’ Committee; protocol number: aLMS/SF/UH/02951(3). Written informed consent from the patients/participants OR patients'/participants' legal guardian/next of kin was not required to participate in this study in accordance with the national legislation and the institutional requirements.

## Author Contributions

FS and AV have conceived the idea of the manuscript and have coordinated the whole project. FN, CZ, DA, and LG have actually carried out the process of both data collection and systematization. FN performed the literature searching, the analysis of data and drafted the manuscript. FS, JC, and AG supervised the manuscript and contributed to the final version of the manuscript. FS approved the final content of the manuscript. JC provided data from the EMCDDA and UNODC databases for the purposes of this research. FS, JC, and AG have provided relevant epidemiological data and have contributed as well to the drafting and checking of the paper itself.

## Conflict of Interest

None of the authors of this paper was directly involved with the website development. AV has conceived the idea of a new early detention software for NPS, which was developed by the professionals at Damicom srl, a small enterprise from Rome (Italy). FS and AV have coordinated the testing of the web crawler. FN, CZ, and DA have suggested minor changes to the software which have made the screening process more precise and efficient.

The remaining authors declare that the research was conducted in the absence of any commercial or financial relationships that could be construed as a potential conflict of interest.

The reviewer, SC, declared a shared affiliation, though no collaboration, with several of the authors, FN, FS, JMC, AG, DA, CZ, and AV to the handling editor.

## Abbreviations

ADHD, attention deficit hyperactivity disorder; CEs, cognitive enhancers; EDND, European Database on New Drugs; EMCDDA, European Monitoring Centre for Drugs and Drug Addiction; EWA, Early Warning Advisory; GABA, gamma-aminobutyric acid; IPEDs, imaging and performance enhancing drugs; MPH, methylphenidate; NPS, novel psychoactive substances; SSRIs, Selective Serotonin Re-uptake Inhibitors; UNODC, United Nations Office on Drugs and Crime.

## Appendix

**Table A1:** Full list of CE identified by NPSfinder^®^ (n=142).

N	NPSfinder^®^ name	Other names	IUPAC (isomerdesign)	EDND (April 2019)	UNODC EWA NPS (July 2019)	Unique to NPSfinder^®^ database	NPSfinder^®^ Brief description
1	123I-Ioflupane	Datscan; Ioflupane (FPCIT); [I-123] N-ω-fluoropropyl- 2β-carbomethoxy- 3β-(4-iodophenyl) nortropane	methyl (1R,2S,3S,5S)-8-(3-fluoropropyl)-3-(4-(123I)iodanylphenyl)-8-azabicyclo[3.2.1]octane-2-carboxylate	N	**N**	**Y**	Phenyltropane isotope used for as a solution to inject into living test subjects for neuroimaging in the diagnosis of Parkinson’s Disease.
2	2C-D	2C-D, 2C-M; 2C-D; 2C-M; 2,5-Dimethoxy-4-methylphenethylamine; LE-25	1-(2,5-Dimethoxy-4-methylphenyl)-2-aminoethane	Y	Y	N	2C-D, or 2,5-dimethoxy-4-methylphenethylamine, is a substituted phenethylamine featuring a phenyl ring bound to an amino (NH2) group through an ethyl chain. 2C-D contains methoxy functional groups CH3O- attached to carbons R2 and R5 as well as a methyl group attached to carbon R4 of the phenyl ring.
3	3,4-Dichloromethylphenidate	3,4-DCMP; 3,4-CTMP	3,4-Dichloromethylphenidate	Y	Y	N	A potent stimulant drug related to methylphenidate. 3,4-DCMP, the threo-diastereomer, is approximately seven times more potent than methylphenidate in animal studies, but has weaker reinforcing effects due to its slower onset of action.
4	4 CTMP	dichloropane	Methyl (2S,3S)-3-(3,4-dichlorophenyl)-8-methyl-8-azabicyclo[3.2.1]octane-2-carboxylate	N	N	Y	Dichloropane (also known as RTI-111 or O-401) is a novel stimulant substance of the tropane class. Stimulant of the Phenyltropane class that acts as a SNDRI. Yet being the Tropane analogue of 3,4-CTMP.
5	4-fluoromethylphenidate	4F-MPH; 4-FMPH; 4FTMP;4F-TMP	Methyl 2-(4-fluorophenyl)-2-(piperidin-2-yl)acetate	Y	Y	N	4-fluoromethylphenidate belongs to the piperidine chemical class and is the 4-fluoro derivative of the internationally controlled substance methylphenidate (Ritalin). It has been advertised online that 4-fluoromethylphenidate was developed as a replacement for ethylphenidate. Dosage indications and duration of effect have also been suggested online.
6	4- Methylmethylphenidate	threo-4-Methylmethylphenidate; 4MeTMP	threo-4-Methylmethylphenidate	Y	Y	N	4-methylmethylphenidate is a ring methylated derivative of the piperidine compound methylphenidate, the active pharmaceutical ingredient in the medicine Ritalin, which is used in the treatment of ADHD. 4-methylmethylphenidate has been researched as a potential cocaine antagonist by blocking the binding of cocaine to the dopamine transporter, when it was reported to be a slightly more potent inhibitor of dopamine uptake, compared to methylphenidate (126 nM vs. 224 nM). [Deutsch 1996] - 4-methylmethylphenidate is compound 1s in this paper, methylphenidate is compound 1a.
7	5-HTP	5-hydroxytryptophan; tryptophan; l-tryptophan; oxitriptan; Oxitriptan; Cincofarm; Levothym; Levotonine; Oxyfan; Telesol; Tript-OH; Triptum	2-amino-3-(5-hydroxy-1H-indol-3-yl)propanoic acid	Y	Y	N	The precursor to serotonin. In some countries it is sold OTC as a supplement for mood stabilisation and insomnia. It is frequently used as a recovery supplement following the use of MDMA or any other drug that depletes serotonin. Do not mix this drug with anything serotonergic, as this can cause serotonin syndrome.
8	Acetildenafil	Hongdenafil	C25H34N6O3	N	N	Y	An RC analogue of sildenafil (Sildenafil) often missold as a hidden ingredient in many ‘natural’ sexual potency blends and supplements.
9	Acetyl-L-carnitine	Tripsitter Party Supplement	(3R)-3-acetyloxy-4-(trimethylazaniumyl)butanoate	N	N	Y	
10	**Adderall**	Adderall XR, and Mydayis	N/A	N	N	Y	Adderall is a psychostimulant drug, it is composed of 4 different amphetamine salts containing Dextroamphetamine and Levoamphetamine. Its 4 different salts are: 1/4th Dextroamphetamine Saccharate, 1/4th Dextroamphetamine Sulfate, 1/4 Racemic Amphetamine Aspartate Monohydrate, and 1/4 Racemic Amphetamine Sulfate.
11	**Adrafinil**	Olmifon; CRL40028	(+-)-2-Benzhydrylsulfinylethanehydroxamic acid	Y	Y	N	Adrafinil is very structurally similar to its close chemical cousin and bioactive metabolite, modafinil. The only structural difference is the that terminal amide hydroxyl group of adrafinil ((diphenylmethyl)sulfinyl-2 acetohydroxamic acid) is lacking in modafinil (diphenylmethyl)sulfinyl-2 acetamide). is a eugeroic that was formerly used in France to promote alertness, attention, wakefulness, mood, and other parameters, particularly in the elderly.[3][4] It was also used off-label by individuals who wished to avoid fatigue, such as night workers or others who needed to stay awake and alert for long periods of time. Additionally, “adrafinil is known to a larger nonscientific audience, where it is considered to be a nootropic agent.
12	**Alpha GPC**	choline alfoscerate; L-Alpha glycerylphosphorylcholine;	[(2R)-2,3-Dihydroxypropyl] 2-trimethylazaniumylethyl phosphate	N	N	Y	Alpha-GPC is a naturally-occurring choline compound found endogenously (naturally) in the brain which is also made and used for oral consumption. Structurally, Alpha-GPC is comprised of a choline group bound to a glycerol molecule *via* a phosphate group.
13	Aminotadalafil	385769-84-6 UNII-FY501QO030 FY501QO030 Amino-tadalafil RR-ATDF Tadalafil-Amino Tadalafil, Amino	(2R,8R)-6-amino-2-(1,3-benzodioxol-5-yl)-3,6,17-triazatetracyclo[8.7.0.03,8.011,16]heptadeca-1(10),11,13,15-tetraene-4,7-dione	N	N	N	An analogue of tadafinil, better known as Tadalafil (Sildenafil). Infamous for being missold in the ‘Alpha Male’ sexual enhancement supplement.
14	**Aniracetam**	methoxybenzoyl)-2-pyrrolidinon; 1-(p-methoxybenzoyl)-2-pyrrolidinone; P-METHOXYBENZOYL-2-PIRROLIDONE; AKOS005066313; Tox21_110086_1; AB04115; ACN-048215; API0001505; BCP9000303; CCG-204210; CS-1793; DB04599; KS-5313; LP00115; NSC-758223; Ro-135057; 2-Pyrrolidinone,1-(4-methoxybenzoyl)-; IDI1_000403;	1-[(4-Methoxybenzoyl)]-2-pyrrolidinone	N	N	Y	Aniracetam is a pyrrolidinone compound of the racetam family, and has an additional anisoyl ring with a methoxy group at the lone para position. (replacing the amine group of piracetam) with an O-methoxy group on the furthest binding point. Its structure is dissimilar to that of oxiracetam (which is quite similar to piracetam) and pramiracetam (a fairly unique structure) Aniracetam is related structurally to nefiracetam.
15	Areca nut	Betel nut; Paans (the combination of Betel leaves, lime, & Areca catechu)	N/A	N	N	Y	The fruit of the Areca catechu palm tree, also known as the “Betel Nut”, contain the stimulant arecoline. Native to SE Asia, the nuts are ground and often combined with mineral lime and wrapped in the leaf of a Betel pepper plant, although they are sometimes consumed buccally (‘chewed’) alone. Notably, frequent use can stain teeth black and its daily use is associated with increased risk of mouth cancers. Variants of the betel and lime combination are extremely common in many Asian cultures and have a long history of human use.
16	Arecoline (transdermal patch)		Methyl 1-methyl-1,2,5,6-tetrahydropyridine-3-carboxylate	Y	Y	N	Arecoline is an alkaloid natural product found in oil form in the areca nut, the fruit of the areca palm (Areca catechu). In some Asian countries, areca nut is chewed along with betel leaf to obtain a stimulating effect.
17	Armodafinil	Nuvigil, Waklert, Artvigil, R-Modawake, Neoresotyl; (R)-Modafinil	2-[(R)-benzhydrylsulfinyl]acetamide	N	N	Y	(R)-Modafinil, or Armodafinil, is a psychoactive molecule of the benzhydryl class. Benzhydryl compounds are comprised of two benzene rings attached to a single carbon molecule. Armodafinil is classified as a sulphinyl benzhydryl molecule, as it also contains a sulphinyl group, a sulfur molecule double-bonded to an oxygen molecule attached to the carbon of the benzhydryl group.
18	Atomoxetine	Strattera	(-)-N-methyl-3-phenyl-3-(o-tolyloxy)-propylamine; (R)-N-methyl-3-phenyl-3-(o-tolyloxy)propan-1-amine	Y	N	N	Atomoxetine is a selective norepinephrine reuptake inhibitor (SNRI) approved as a less stimulating treatment for ADHD in 2002 (U.S.). The precise mechanism by which it produces its therapeutic effects is unknown, but is thought to be related to its SNRI action. It appears to have minimal affinity for noradrenergic receptors or other neurotransmitter transporters or receptors.
19	B-HO-Hordenine		4-[2-(Dimethylamino)-1-hydroxyethyl]phenol	N	N	Y	Hordenine (N,N-dimethyltyramine) is an alkaloid of the phenethylamine class that occurs naturally in a variety of plants, taking its name from one of the most common, barley (Hordeum species). Chemically, hordenine is the N-methyl derivative of N-methyltyramine, and the N,N-dimethyl derivative of the well-known biogenic amine tyramine, from which it is biosynthetically derived and with which it shares some pharmacological properties
20	Bacopa monnieri	Omnimind; Paneuromix	N/A	N	N	Y	It stimulates your grey matter and kicks it up a notch, leaving you more time to enjoy the good things in life.
21	Benzyl cyanide	2-Phenylacetonitrile; α-Tolunitrile; Benzylnitrile	2-Phenylacetonitrile	N	N	Y	Benzyl cyanide is a useful precursor to numerous pharmaceuticals. Examples include: Anorectics (e.g. sibutramine)
22	Beta-asarone	Cis-Isoasarone; (Z)-Asarone; Cis-Asarone; cis-2,4,5-Trimethoxyphenylprop-1-ene	1,2,4-Trimethoxy-5-[(1Z)-prop-1-en-1-yl]benzene	N	N	Y	It is one of the two isomers of Asarone, a chemical compound of the phenylpropanoid class found in certain plants such as Acorus and Asarum. It is no clear if can be metabolized to trimethoxyamphetamine.
23	Caffeine	Caffeine, Vivarin, Cafcit, Alert; Caffeine; Vivarin; Cafcit; Alert;1,3,7-trimethylxanthine, methyltheobromine	1,3,7-trimethylpurine-2,6-dione	N	N	Y	Caffeine is an alkaloid with a substituted xanthine core. Xanthine is a substituted purine comprised of two fused rings: a pyrimidine and an imidazole. Pryimidine is a six-membered ring with nitrogen constituents at R1 and R3; imidazole is a 5 membered ring with nitrogen substituents at R1 and R3. Xanthine contains oxygen groups double-bonded to R2 and R6.
24	Catechins	Green tea extract	(2S,3R)-2-(3,4-dihydroxyphenyl)-3,4-dihydro-2H-chromene-3,5,7-triol	N	N	Y	Green tea (Camellia sinensis) plays an important role in the traditional Chinese herbal medicine. Immediately after harvesting the green tea leaves are steamed and dried instead of fermented, so the bioactive ingredients remains preserved optimally.
25	Celastrus paniculatus	Black oil plant seeds	N/A	N	N	Y	Celastrus paniculatus, also known as Black oil plant, climbing staff tree and intellect tree is a woody, fruit-bearing vine from India. Black oil plant seeds are used in Ayurvedic medicine due to their varied medicinal properties. Celastrus paniculatus’ ability to protect the brain and to improve memory functions makes it an effective nootropic.
26	**Centrophenoxine**	Meclofenoxate; Amipolen; Analux; Brenal; Cellative; Centrophenoxin; Cerebron; Cerutil; Closete; Helfergin; lucidril; Lucidryl; Lutiaron; Marucotol; Proserout; Proseryl; Ropoxyl	2-Dimethylaminoethyl (4-chlorophenoxy)acetate	N	N	Y	Meclofenoxate is broken down by the liver into DMAE and PCPA (parachlorphenoxyacetic acid). It has been shown to cause mild memory improvement in people with dementia and has been marketed as an anti-aging supplement.
27	**Choline Bitartrate**	CHOLINE BITARTRATE; 87-67-2; CHOLINI BITARTRAS; 2-Hydroxy-N,N,N-trimethylethanaminium 3-carboxy-2,3-dihydroxypropanoate; 2-(Hydroxyethyl)trimethylammonium bitartrate; Choline bitartrate, 97%; Choline tartrate (1:1); and many others.	2-Hydroxy-N,N,N-trimethylethan-1-aminium	N	N	Y	Choline is comprised of a quaternary ammonium group and an alcohol functional group, which are connected through an ethyl chain. Its charged cation can bind to a negative group or atom to form various salts, which can produce varying effects. Choline chloride can form a low-melting deep eutectic solvent mixture with urea with unusual properties.
28	Cinnamon extract		2-methoxy-4-prop-2-enylphenol;[(E)-prop-1-enyl]benzene	N	N	Y	
29	**Citicoline**	Neurocoline; cytidine diphosphate-choline; CDP-Choline; cytidine 5’-diphosphocholine	(2R,3S,4R,5R)-5-(4-amino-2-oxopyrimidin-1-yl)-3,4-dihydroxyoxolan-2-ylmethoxy-hydroxyphosphoryl 2-(trimethylazaniumyl)ethyl phosphate	N	N	Y	Citicoline, or cytidine diphosphate-choline, is a naturally occurring substance found in human cell tissue and synthesized as a sodium salt as a supplement. Its chemical structure is comprised of a cytidine nucleoside attached to a choline group through a diphosphate bridge. Citicoline is a chemical intermediary in the biosynthesis of phosphatidylcholine, a major phospholipid in cell membranes.
30	Coluracetam	acetoamide; AJ-08232; DS-14004; HY-17553; AX8209310; KB-271979; ST2407347; TC-072260; 4CH-017490; FT-0697594; Y1294; 463C819; J-690145; I14-13061; High-affinity choline uptake facilitator (CNS disorders), Mitsubishi; High-affinity choline uptake facilitator (depression/anxiety), BrainCells; Neurons growth promoting compound (major depressive disorder/anxiety), BrainCells; 1-Pyrrolidineacetamide, 2-oxo-N-(5,6,7,8-tetrahydro-2,3-dimethylfuro(2,3-b)quinolin-4-yl)-; N-(2,3-dimethyl-5,6,7,8-tetrahydrofuro[2,3-b]quinolin-4-yl)-2-(2-oxo-1-pyrrolidinyl)acetamide and many others.	N-(2,3-Dimethyl-5,6,7,8-tetrahydrofuro[2,3-b]quinolin-4-yl)-2-(2-oxo-1-pyrrolidinyl)acetamide	N	N	Y	Coluracetam, or N-(2,3-Dimethyl-5,6,7,8-tetrahydrofuro[2,3-b]quinolin-4-yl)-2-(2-oxo-1-pyrrolidinyl)acetamide, is a synthetic compound of the racetam family. Racetams share a pyrrolidine nucleus, a five-member nitrogenous ring with a ketone bonded oxygen at R2.[2] This 2-pyrrolidone ring is bound to the terminal carbon of an acetamide group, an ethyl amide chain with a ketone bond (C=O) at the alpha carbon.
31	Coumarins	Leonotis Leonurus flowers; Monnier’s snow parsley seeds; Leonotis Leeonorus 20X extract; wild lettuce	chromen-2-one	N	N	Y	Ingredient of wild lettuce. Biennial, growing up to 2-4 feet, maximum height of 6 feet (cultivated plants usually smaller). The erect stem, springing from a brown tap-root, is smooth and pale green, sometimes spotted with purple. Leaves are from 6 to 18 inches long, flowers are pale yellow, with large open clusters.
32	**Creatine**	N-Carbamimidoyl-N-methylglycine, Methylguanidoacetic acid; Creatine; N-Carbamimidoyl-N-methylglycine; Methylguanidoacetic acid	2-[Carbamimidoyl(methyl)amino]acetic acid	N	N	Y	Creatine is a nitrogenous amino acid produced endogenously and synthesized for consumption. Synthetic creatine is usually made from sarcosine (or its salts) and cyanamide which are combined in a reactor with catalyst compounds. The reactor is heated and pressurized, causing creatine crystals to form. The crystalline creatine is then purified by centrifuge and vacuum dried. The dried creatine compound is milled into a fine powder for improved bioavailability. Milling techniques differ, resulting in final products of varying solubility and bioavailability. For instance, creatine compounds milled to 200 mesh are referred to as micronized
33	Curcumin	Turmeric tea golden ginger; Turmeric drink golden cocoa	(1E,6E)-1,7-bis(4-hydroxy-3-methoxyphenyl)hepta-1,6-diene-3,5-dione	N	N	Y	Turmeric and ginger do not only look like one another; they also fit together very well! That’s proven with this excellent, beneficial tea by TAKA Turmeric with Golden Ginger. The delicious organic blend of turmeric and ginger provides a warming and invigorating effect. The black pepper and coconut increase the effect of the active ingredient in turmeric: Curcumin. Do not wait any longer for the perfect turmeric tea: order Golden Ginger.
34	Dehydroepiandrosterone	DHEA; androstenolone; Prasterone	3S,8R,9S,10R,13S,14S)-3-hydroxy-10,13-dimethyl-1,2,3,4,7,8,9,11,12,14,15,16-dodecahydrocyclopenta[a]phenanthren-17-one	N	N	Y	DHEA supplements are said to increase energy, enhance memory and cognitive function, improve immunity, and reduce the effects of stress. Dehydroepiandrosterone (DHEA), also known as androstenolone, is an endogenous steroid hormone. DHEA also has a variety of potential biological effects in its own right, binding to an array of nuclear and cell surface receptors, and acting as a neurosteroid and neurotrophin. In addition to its role as a natural hormone, DHEA is used as an over-the-counter supplement and medication; for information on DHEA as a supplement or medication
35	Deoxypipradol	2-diphenylmethylpiperidine; 2-DPMP; 2-Diphenylmethylpiperidine 2-benzhydrylpiperidine 519-74-4 Desoxypipradrol 2-(Diphenylmethyl)piperidine Piperidine, 2-(diphenylmethyl)- Piperidine,2-(diphenylmethyl)- 2-DPMP AK-24338 2-(Diphenylmethyl)piperidine; 2-Benzhydrylpiperidine; Desoxypipradol; Ivory Wave	2-diphenylmethylpiperidine	Y	Y	N	Desoxypipradrol, acts as a norepinephrine-dopamine reuptake inhibitor (NDRI) developed by Ciba in the 1950s. Desoxypipradrol is closely related on a structural level to the compounds methylphenidate and pipradrol, all three of which share a similar pharmacological action.2-DPMP is a powerful stimulant that has been found in the product ‘Ivory Wave’. It was taken as a ‘legal high’ and has amphetamine-like stimulant effects similar to speed. 2-DPMP effects can be both powerful and long-lasting, with effects that can last as long as 5-7 days - some users have had to go to hospital for help.
36	Dexanabinol	ETS2101; Dexanabinol; (6aS,10aS)-9-(Hydroxymethyl)-6,6-dimethyl-3-(2-methyloctan-2-yl)-6a,7,10,10a-tetrahydro-6H-dibenzo[b,d]pyran-1-ol	(6aS,10aS)-9-(Hydroxymethyl)-6,6-dimethyl-3-(2-methyloctan-2-yl)-6a,7,10,10a-tetrahydro-6H-dibenzo[b,d]pyran-1-ol	N	N	Y	Cannabinoid
37	Dexmethylphenidate	D-threo-Methylphenidate; Methyl D-phenidate; Focalin; UNII-M32RH9MFGP; D-Methylphenidate; 40431-64-9; D-TMP; M32RH9MFGP; CHEMBL827; CHEBI:51860; Focalin; methyl (R)-phenyl[(R)-piperidin-2-yl]acetate; D-MPH; Attenade; dexmetilfenidato; AC1L4BP3; SCHEMBL34326; GTPL7554; DUGOZIWVEXMGBE-CHWSQXEVSA-N; ZINC896711; 2-Piperidineacetic acid, alpha-phenyl-, methyl ester, (alphaR,2R)	Methyl (2R)-phenyl[(2R)-2-piperidinyl]acetate	N	N	Y	Dexmethylphenidate, sold under the trade names Focalin among others, is a central nervous system (CNS) stimulant used in the treatment of attention deficit hyperactivity disorder (ADHD) and narcolepsy.It is in the phenethylamine and piperidine classes of medications. It is the active dextrorotatory enantiomer of methylphenidate.
38	**Dextroamphetamine**	Dexedrine; Metamina; Attentin; Zenzedi; Procentra; Amfexa; D-Amphetamine; Dextroamphetamine sulphate; Dexamfetamine; Dexamphetamine	(2S)-1-Phenylpropan-2-amine	N	N	Y	Dextroamphetamine is a potent central nervous system (CNS) stimulant and amphetamine enantiomer that is prescribed for the treatment of attention deficit hyperactivity disorder (ADHD) and narcolepsy. It is also used as an athletic performance and cognitive enhancer, and recreationally as an aphrodisiac and euphoriant. Dextroamphetamine was also used by military air, tank and special forces as a ‘go-pill’ during fatigue-inducing missions such as night-time bombing missions or extended combat operations. The amphetamine molecule exists as two enantiomers (in other words, two different molecules that are mirror images of one another), levoamphetamine and dextroamphetamine. Dextroamphetamine is the more active, dextrorotatory, or ‘right-handed’, enantiomer of the amphetamine molecule. Pharmaceutical dextroamphetamine sulfate is available as both a a brand name and generic drug in a variety of dosage forms.
39	Dimethylaminoethanol	DMAE; deanol; di-methyl-amino-ethanol; DMEA	2-(Dimethylamino)ethanol	N	N	Y	DMAE is a precursor of choline and an anti-oxidant that is found naturally in the brain. It is said to improve memory and learning as well as increasing ability to concentrate.
40	DL-Phenylalanine	DLPA; 2-amino-3-phenylpropanoic acid	2-amino-3-phenylpropanoic acid	N	N	Y	DL-Phenylalanine is a racemic mixture of phenylalanine, an aromatic amino acid with antidepressant, analgesic and appetite suppressant properties. The antidepressant effect of DL-phenylalanine may be accounted for by its precursor role in the synthesis of the neurotransmitters norepinephrine and dopamine. Elevated brain norepinephrine and dopamine levels are thought to be associated with antidepressant effects. This agent also plays a role in alleviating mood swings of premenstrual syndrome (PMS), increasing energy and mental alertness and heighten the ability to focus in individuals with attention deficit hyperactivity disorder (ADHD).
41	Drinabant	AVE-1625	N-{1-[Bis(4-chlorophenyl)methyl]azetidin-3-yl}-N-(3,5-difluorophenyl)methanesulfonamide	N	N	Y	AVE-1625 is a highly potent, selective antagonist for the CB1 receptor with Ki values of 0.16-0.44 nM. Drinabant reached phase IIb clinical trials as obesity treatment in the treatment of obesity but was shortly thereafter discontinued, likely due to the observation of severe psychiatric side effects including anxiety, depression, and thoughts of suicide in patients treated with the now-withdrawn rimonabant, another CB1 antagonist that was also under development by Sanofi-Aventis.
42	Dronabinol	ultrapure THC; Namisol; dronabinol; Marinol; syndros; cesamet; δ9-thc; δ9–tetrahydrocannabino; delta9-tetrahydrocannabino; delta9-thc	(−)-trans-Δ^9^-tetrahydrocannabinol	N	N	Y	Dronabinol – trade names Marinol and Syndros – is a synthetic form of tetrahydrocannabinol (THC) approved by the FDA as an appetite stimulant for people with AIDS and antiemetic for people receiving chemotherapy. Synthetically created D9-THC, the main psychoactive ingredient in Cannabis.
43	Ephedrine	Ephedrine Erythro Isomer; Ephedrine Hydrochloride; Ephedrine Renaudin; Ephedrine Sulfate; Erythro Isomer of Ephedrine; Hydrochloride, Ephedrine; Renaudin, Ephedrine; Sal Phedrine; Sal-Phedrine; SalPhedrine; Sulfate, Ephedrine; Mini Thins; Sudafed; Trucker’s Speed; (-)-alpha-(1-methylaminoethyl)benzyl alcohol	(1R,2S)-2-(methylamino)-1-phenylpropan-1-ol	N	N	Y	Ephedrine is the active ingredient in Ephedra sinica. One isomer (pseudoephedrine) is widely sold as a decongestant while the other (ephedrine) is a commonly used stimulant. Ephedrine is a medication and stimulant. It is often used to prevent low blood pressure during spinal anesthesia. It has also been used for asthma, narcolepsy, and obesity but is not the preferred treatment. It is of unclear benefit in nasal congestion. As a phenethylamine, ephedrine has a similar chemical structure to amphetamines and is a methamphetamine analogue having the methamphetamine structure with a hydroxyl group at the β position.
44	Ethylphenidate	EPH	Ethyl 2-phenyl-2-(piperidin-2-yl)acetate	Y	Y	N	Ethylphenidate is a synthetic molecule of the substituted phenethylamine class. It contains a phenethylamine core featuring a phenyl ring bound to an amino -NH2 group through an ethyl chain. It is structurally similar to amphetamine, featuring a substitution at Ra which is incorporated into a piperdine ring ending at the terminal amine of the phenethylamine chain. Additionally, it contains an ethyl acetate bound to R2 or its structure. Ethylphenidate is structurally differed to methylphenidate by elongation of the carbon chain. Ethyl- regards the side chain of two carbon atoms, phen- indicates the phenyl ring, id- is contracted from a piperidine ring, and -ate indicates the acetate group containing the oxygens. Ethylphenidate is a chiral compound, presumably produced as a racemic mixture.
45	**F-Phenibut**	Fluorophenibut; Fluorobut; b-(4-flourophenyl)-GABA	5-amino-4-(4-fluorophenyl)-1-hydroxypentan-2-one	N	N	Y	As with phenibut, F-Phenibut is a derivative of GABA, except with a fluorine-substituted phenyl group in the b-position of the molecule. It is a chiral molecule and thus has two potential configurations as (R)- and (S)-enantiomers.[4] It has an almost identical chemical structure to baclofen (only replacing a chlorine with a fluorine atom in the para-position of the phenyl group).[5] Additionally, the widely prescribed gabapentinoid pregabalin also possesses a similar structure as phenibut, except that the phenyl group is instead replaced with an isobutyl group.
46	**Fasoracetam**	NS-105; LAM-105; (5R)-5-oxo-D-prolinepiperidinamide monohydrate; NS-105; AEVI-001; LAM 105; MDGN-001; NFC 1	(5R)-5-(piperidine-1-carbonyl) pyrrolidin-2-one	N	N	Y	A substance in the racetam family. Appears to be a GABA(B) agonist, and has shown to block memory disruptions caused by Baclofen, another GABA(B) Agonist. Similar to another compound in the racetam family Coluracetam, it enhances High affinity choline reuptake (HACU). It is a nootropic, and has been in clinical trials for vascular dementia and attention deficit hyperactivity disorder
47	**Fipexide**	Attentil; Vigilor; BP 662	1-(1,3-benzodioxol-5-ylmethyl)-4-[(4-chlorophenoxy)acetyl]piperazine	N	N	Y	Fipexide is a mild stimulant that is thought to indirectly affect dopamine levels in the brain. It is said to improve short term memory, attention, learning, and cognition. There is a risk of serious liver damage and high fever with use. Fipexide (Attentil, Vigilor) is a psychoactive drug of the piperazine chemical class which was developed in Italy in 1983. It was used as a nootropic drug in Italy and France, mainly for the treatment of senile dementia, but is no longer in common use due to the occurrence of rare adverse drug reactions including fever and hepatitis
48	Fladrafinil	CRL 40941; Fluorafinil; fluoromodafinil	4;4-difluorobenzhydrylsulfinylacetohydroxamic acid	Y	Y	N	Fladrafinil was described in a patent by Louis Lafon Laboratories in the 1980’s, the same company that developed the atypical psychostimulant adrafinil (CRL 40028) in the 1970’s. Fladrafinil is the 4,4’-difluoro derivative of adrafinil. A substance closely related to Adrafinil and Modafinil. It is the bis(p-fluoro) ring derivative of Adrafinil.
49	Flavonoids		2-phenylchromen-4-one	N	N	Y	Ingredients of wild Lettuce. Biennial, growing up to 2-4 feet, maximum height of 6 feet (cultivated plants usually smaller). The erect stem, springing from a brown tap-root, is smooth and pale green, sometimes spotted with purple. Leaves are from 6 to 18 inches long, flowers are pale yellow, with large open clusters.
50	Flmodafinil	lauflumide; bisfluoromodafinil; RL-40-940	C15H13F2NO2S	Y	N	N	Bisfluoro analogue Modafinil. Has been sold online as a research chemical. Was patented in 2013. Is slightly more potent than Armodafinil. CRL-40,940 (also known as flmodafinil, bisfluoromodafinil and lauflumide) is a selective dopaminergic reuptake inhibitor, and is the bisfluoro analog of the eugeroic modafinil and has been sold online as a designer drug.
51	GABA	γ-aminobutyric acid; gamma aminobutyric acid	4-Aminobutanoic acid	N	N	Y	Gaba is an inhibitory neurotransmitter found naturally in the brain. Research suggests that increased levels of gaba might help reduce the mental decline associated with aging.GABA is sold as a dietary supplement.
52	Galantamine	Razadyne; Reminyl; Nivalin; Razadyne ER; Reminyl; Lycoremine	(4aS,6R,8aS)-5,6,9,10,11,12-Hexahydro-3-methoxy-11-methyl-4aH-[1]benzofuro[3a,3,2-ef][2]benzazepin-6-ol	N	N	Y	Galantamine is a complex alkaloid that is found endogenously in certain plants and synthesized for medical use. It is comprised of a fusion between a methoxy substituted benzene ring to a hydrogenated and methylated azepine ring along with a hydroxylated benzofuran group.
53	Geranamine	DMAA; methylhexanamine; methylhexamine; geranamine; geranium extract; geranium oil; 2-amino-4-methylhexane; dimethylamylamine; DMAA; 1,3-dimethylamylamine; 1,3-DMAA; 1,3-dimethylpentylamine; 4-methyl-2-hexanamine; 4-methyl-2-hexylamine; orthan; Forthane; Floradrene;	4-methylhexan-2-amine	Y	N	N	Also known as methylhexanamine, this sympathetomimetic drug was developed as a nasal decongestant by Eli Lilly in the 1940s. It has been used as a weight loss aid and missold as a dietary supplement and component of some energy drinks. Carries a risk of heart attack, stroke and other life-threatening cardiovascular issues. It may occur naturally as a component of the oil extracted from the geranium plant.
54	Ginger root extracts		(E)-1-(4-hydroxy-3-methoxyphenyl)dec-4-en-3-one;1-(4-hydroxy-3-methoxyphenyl)-5-methyldecan-3-one	N	N	Y	Ginger is an herbaceous tropical perennial grows 2-4 feet tall from an aromatic, tuberous root. Leaves are grass-like and 6-12 inches long. Flowers are dense, red and yellow cone-like spikes 3 inches long at the end of a 6-12 inch stalk.
55	Gingko biloba	Gingko biloba powder	N/A	N	N	Y	Ginkgo biloba contains ginkgolides that have a positive effect on blood circulation and oxygen levels, which are associated with brain performance and help maintain cognitive function. Ginkgo contributes to a clear mind and mental focus. The brain boosting effects of ginkgo can help improve memory and memory recall, whether you’re preparing for an exam or simply want to keep your mind sharp. Effect Ginkgo has stimulating properties. It has a positive effect on cognitive function and mental alertness. Ginkgo helps maintain healthy blood vessels.
56	Ginseng	Tartar Root; Five-fingers	bis[3,4,5-trihydroxy-6-(hydroxymethyl)oxan-2-yl] 2,3-dihydroxy-6b-(hydroxymethyl)-4,6a,11,11,14b-pentamethyl-1,2,3,4a,5,6,7,8,9,10,12,12a,14,14a-tetradecahydropicene-4,8a-dicarboxylate	N	N	Y	Ginseng’s thin, single stem grows from a bud at the top of the root that rises and separates into a whorl of compound leaves. Small green flowers radiate, umbrella-like from the end of a stalk and are eventually replaced by red berries.
57	Harmaline		7-methoxy-1-methyl-4,9-dihydro-3H-pyrido[3,4-b]indole	N	Y	N	Harmaline is a fluorescent psychoactive indole alkaloid from the group of harmala alkaloids and beta-carbolines. It is the partially hydrogenated form of harmine. Various plants contain harmaline including Peganum harmala (Syrian rue) as well as the hallucinogenic beverage ayahuasca, which is traditionally brewed using Banisteriopsis caapi. Present at 3% by dry weight, the harmala alkaloids may be extracted from the Syrian rue seeds.
58	Harmalol		1-Methyl-4,9-dihydro-3H-pyrido[3,4-b]indol-7-ol	N	N	Y	Harmalol is a bioactive beta-carboline and a member of the harmala alkaloids.
59	Homosildenafil	methyl-sildenafil	C23H32N6O4S	N	N	Y	An analogue of sildenafil (Sildenafil) with similar effects. Has been missold in certain ‘herbal’ blends and dietary supplements for sexual potency. Little is known about the pharmacology or safety profile of this drug in humans, potentially less potent than sildenafil.
60	Hordenine	4-Hydroxy-N,N-dimethylphenethylamin	4-(2-Dimethylaminoethyl)phenol	N	N	Y	Hordenine (N,N-dimethyltyramine) is an alkaloid of the phenethylamine class that occurs naturally in a variety of plants, taking its name from one of the most common, barley (Hordeum species). Chemically, hordenine is the N-methyl derivative of N-methyltyramine, and the N,N-dimethyl derivative of the well-known biogenic amine tyramine, from which it is biosynthetically derived and with which it shares some pharmacological properties.
61	Huperzine A	Amino-13-ethylidene-11-methyl-6-aza-tricyclo[7.3.1.0*2,7*]trideca-2(7),3,10-trien-5-one(Huperzine A); (-)1-Amino-13-ethylidene-11-methyl-6-aza-tricyclo[7.3.1.0*2,7*]trideca-2(7),3,10-trien-5-one((-)-Huperzine A);	(1R,9R,13E)-1-Amino-13-ethylidene-11-methyl-6-azatricyclo[7.3.1.0^2^,^7^]trideca-2(7),3,10-trien-5-one	N	N	Y	A compound that is extracted from the herbs of Huperziceae family. Is known as an acetylcholinesterase inhibitor, which stops an enzyme from breaking down acetylcholine which results in increases in acetylcholine. Is currently in preliminary trials for Alzheimer’s.
62	Hydergine	Gerimal; Niloric; co-dergocrine mesilate; dihydroergotoxine mesylate; Ergoloid mesylates	(6aR,9R,10aR)-N-[(1S,2S,4R,7S)-2-hydroxy-5,8-dioxo-4,7-di(propan-2-yl)-3-oxa-6,9-diazatricyclo[7.3.0.02,6]dodecan-4-yl]-7-methyl-6,6a,8,9,10,10a-hexahydro-4H-indolo[4,3-fg]quinoline-9-carboxamide;methanesulfonic acid	N	N	Y	Ergoloid mesylates are ergot alkaloids which act as neuroprotective anti-oxidants. They are also said to increase blood flow to the brain and generally increase cognitive abilities though the evidence is contradictory on these points. It was approved by the FDA in 1951.
63	Icariin	Horny goat weed extract 10% Icariin	5-hydroxy-2-(4-methoxyphenyl)-8-(3-methylbut-2-enyl)-7-[(2S,3R,4S,5S,6R)-3,4,5-trihydroxy-6-(hydroxymethyl)oxan-2-yl]oxy-3-[(2S,3R,4R,5R,6S)-3,4,5-trihydroxy-6-methyloxan-2-yl]oxychromen-4-one	N	N	Y	Horny goat weed extract 10% Icariin | 3 g - Epimedium
64	Isopropylphenidate	IPH, IPPH; IPPD	Propan-2-yl 2-phenyl-2-(piperidin-2-yl)acetate	Y	Y	N	Isopropylphenidate is a synthetic molecule of the substituted phenethylamine and piperidine classes. It contains a phenethylamine core featuring a phenyl ring bound to an amino (NH2) group *via* an ethyl chain. It is structurally similar to amphetamine, featuring a substitution at Ra which is then incorporated into a piperidine ring ending at the terminal amine of the phenethylamine chain. Additionally, it contains an isopropyl acetate bound to R2 of its molecular structure, a noticeable departure from methylphenidate, which contains a methyl group in this position.
65	JZL-184		4-Nitrophenyl 4-[bis(2H-1,3-benzodioxol-5-yl)(hydroxy)methyl]piperidine-1-carboxylate	N	N	Y	Cannabinoid
66	Kaempferol	Blue Lotus 20X Extract; Blue Lotus tincture 15X Extract; Kratom X Blue Lilly Liquid; Trichocereus Bridgesii Cutting; Trichocereus Bridgesii Monstruosus Cutting	3,5,7-trihydroxy-2-(4-hydroxyphenyl)chromen-4-one	N	N	Y	Blue Lotus flowers have also yielded a variety of alkaloids, including kaempferol, which has mild MAOI properties. Traditionally used as a narcotic, Nymphaea caerulea helps strengthen the male erection. Blue Lily also contains powerful antioxidants. This versatile plant has a lot to offer. Effect Blue Lotus has calming effects. The main effect of the Blue Lotus 20X extract is sedative, calming and relaxing. Blue Lotus offers a very mild, mind opening (hallucinogenic) experience. This potent extract combines very well with red wine, bringing you into a euphoric and ecstatic state. Last but not least, Blue Lotus has aphrodisiac qualities.
67	Kava Kava	Kava; Piper methysticum; Kawa; Awa; Waka; Lawena; Sakau; Yaqona; Kaffa kaffa;	6-[(E)-2-(cyclohexa-1,5-dien-1-yl)ethenyl]-4-methoxy-5,6-dihydro-2H-pyran-2-one	N	N	Y	Kava is a tropical evergreen shrub with large heart-shaped leaves and woody stems. Its thick roots are mashed or ground and made into a cold beverage used similarly to alcohol. It has a long history of ritual and recreational use in Pacific Polynesia and is now a common herbal product.
68	Kratom	Mitragyna speciosa; krath`m (Thai); ketum; kratum;	Methyl (E)-2-[(2S,3S,7aS,12bS)-3-ethyl-7a-hydroxy-8-methoxy-2,3,4,6,7,12b-hexahydro-1H-indolo[2,3-a]quinolizin-2-yl]-3-methoxyprop-2-enoate	Y	N	N	Kratom is the common name for the plant Mitragyna speciosa Korthals. It is a tree indigenous to Southeast Asia (Thailand, northern Malay Peninsula to Borneo); it is mostly grown in the central and southern regions of Thailand, and only rarely in the northern part. There are more than 40 compounds in the leaves of M. multi-solvent, including many indole alkaloids such as mitragynine (once thought to be the primary active constituent), mitraphylline, and 7-hydroxymitragynine (which is currently the most likely candidate for the primary active chemical in the plant). Other active chemicals in M. specials include raubasine, rhynchophylline, and corynantheidine, among many others.
69	**L-655,708**	9H-Imidazo[1,5-a]pyrrolo[2,1-c][1,4]benzodiazepine-1-carboxylic acid, 11,12,13,13a-tetrahydro-7-methoxy-9-oxo-, ethyl ester, (13aS)-; Ethyl (13aS)-7-methoxy-9-oxo-11,12,13,13a-tetrahydro-9H-imidazo[1,5-a]pyrrolo[2,1-c][1,4]benzodiazepine-1-carboxylate; 130477-52-0; CHEMBL52030;	Ethyl 7-methoxy-9-oxo-11,12,13,13a-tetrahydro-9H-imidazo[1,5-a]pyrrolo[2,1-c][1,4]benzodiazepine-1-carboxylate	N	N	Y	It is a nootropic drug invented in 1996 by a team working for Merck, Sharp and Dohme, that was the first compound developed which acts as a subtype-selective inverse agonist at the α5 subtype of the benzodiazepine binding site on the GABAA receptor. It acts as an inverse agonist at the α1, α2, α3 and α5 subtypes, but with much higher affinity for α5, and unlike newer α5 inverse agonists such as α5IA, L-655,708 exerts its subtype selectivity purely *via* higher binding affinity for this receptor subtype, with its efficacy as an inverse agonist being around the same at all the subtypes it binds to.A radiolabelled form of L-655,708 was used to map the distribution of the GABAA α5 subtype in the brain, and it was found to be expressed predominantly in the hippocampus, an area of the brain involved with learning and memory. Activation of this subtype is thought to be largely responsible for producing the cognitive side effects displayed by many benzodiazepine and nonbenzodiazepine drugs, such as amnesia and difficulties with learning and memory, and so this led researchers to conclude that a drug acting as an inverse agonist at this subtype should have the opposite effect and enhance learning and memory.
70	L-Tryptophan	L-TRP	(2S)-2-amino-3-(1H-indol-3-yl)propanoic acid	N	N	Y	Tryptophan (symbol Trp or W) is an α-amino acid that is used in the biosynthesis of proteins. Tryptophan contains an α-amino group, an α-carboxylic acid group, and a side chain indole, making it a non-polar aromatic amino acid. It is essential in humans, meaning the body cannot synthesize it: it must be obtained from the diet. Tryptophan is also a precursor to the neurotransmitter serotonin, the hormone melatonin and vitamin B3. It is encoded by the codon UGG.
71	**Lemon Balm**		N/A	N	N	Y	Lemon Balm is a perennial (its root survives the winter) herb usually growing 1-2 feet (50 cm) tall. The leaves are ovate to heart-shaped and mint-like. Its flowers are white to yellowish in loose, small bunches and have a lemony-minty smell and flavor. Lemon Balm is used in foods and teas, as an insect repellant, and there is evidence that it has anxiolytic, sedative, mood improving, and nootropic effects. Some people report distinct psychoactive effects taking it as a tea, snorting, or smoking it, sometimes in combination with other plants or drugs, though reports are inconsistent.
72	Lisdexamfetamine	lisdextroamfetamine; lisdexamphetamine; Tyvense; Elvanse; Aduvanz; Venvanse; Vyvanse	(2S)-2,6-diamino-N-[(2S)-1-phenylpropan-2-yl]hexanamide	N	N	Y	This drug is a CNS stimulant often prescribed for ADHD, narcolepsy and obesity. It is also a pro-drug for dextroamphetamine, and functions as a method for providing extended-release stimulation. It is sometimes prescribed alongside an SSRI for depression. Lisdexamfetamine (contracted from L-lysine-dextroamphetamine) is a prodrug of the central nervous system (CNS) stimulant dextroamphetamine, a phenethylamine of the amphetamine class that is used in the treatment of attention deficit hyperactivity disorder (ADHD) and binge eating disorder. Its chemical structure consists of dextroamphetamine coupled with the essential amino acid L-lysine. Lisdexamfetamine itself is inactive prior to its absorption and the subsequent rate-limited enzymatic cleavage of the molecule’s L-lysine portion, which produces the active metabolite (dextroamphetamine).
73	Lobeline	derived from lobelia inflata	2-[(2R,6S)-6-[(2S)-2-hydroxy-2-phenylethyl]-1-methylpiperidin-2-yl]-1-phenylethanone	N	N	Y	Native Americans used lobelia to treat respiratory and muscle disorders, and as a purgative. The species used most commonly in modern herbalism is Lobelia inflata (Indian tobacco). However, there are adverse effects that limit the use of lobelia. Lobelia has been used as “asthmador” in Appalachian folk medicine. Two species, L. siphilitica and L. cardinalis, were once considered a cure for syphilis. Herbalist Samuel Thomson popularized medicinal use of lobelia in the United States in the early 19th century, as well as other medicinal plants like goldenseal. One species, Lobelia chinensis is used as one of the fifty fundamental herbs in traditional Chinese medicine. Several studies show that lobelia is ineffective in helping people to quit smoking.
74	Lovegra		N/A	N	N	Y	Lovegra 100 mg, the pink female equivalent to the blue Viagra pill. Specificly developed for women, The Lovegra pill improves blood flow in the genital area. For more intense sexual satisfaction for women, will increase your pleasure and the vaginal moisture is ensured during the entire sex. Did you already discovered Lovegra? If not … you have to try this!!!
75	Maca	Peruvian ginseng; Lepidium meyenii; maca-maca; maino; ayak chichira; ayak willku. Macaridine.	N/A	N	N	Y	Maca is an edible herbaceous biennial plant of the family Brassicaceae that is native to South America in the high Andes mountains of Peru. It is mostly used for sexual and fertility problems.
76	Marijuana	Indian hemp; marijuana; Cannabis, Marijuana, Weed, Pot, Mary Jane, Grass, Herb, Devil’s Lettuce, Jazz Tobacco; Cannabis; Marijuana; Weed; Pot; Mary Jane; Grass; Herb; Devil’s Lettuce; Jazz Tobacco	N/A	N	N	Y	Cannabis is an annual herbaceous flowering plant indigenous to eastern Asia but now of cosmopolitan distribution due to widespread cultivation. It has been cultivated throughout recorded history, used as a source of industrial fibre, seed oil, food, recreation, religious and spiritual moods and medicine. Each part of the plant is harvested differently, depending on the purpose of its use. The species was first classified by Carl Linnaeus in 1753
77	Melatonin	N-Acetyl-5-methoxytryptamine	N-[2-(5-Methoxy-1H-indol-3-yl)ethyl]acetamide	N	N	Y	Melatonin is comprised of a monoamine chain attached to an indole ring at the third carbon. A monoamine chain is made up of an amine group attached to an ethane chain. This monoamine chain can be found in many neurotransmitters, including histamine, dopamine, adrenaline, and noradrenaline. It is also found in many psychoactive substances such as members of the tryptamine and phenethylamine chemical classes.
78	Memantine	Axura, Ebixa, Namenda, Memary	3,5-Dimethyladamantan-1-amine	Y	Y	N	Memantine or 3,5-dimethyladamantan-1-amine is a synthetic molecule classified as a substituted adamantane derivative. Its core structure is adamantane, a diamondoid of four interlocked cyclohexane rings in a stable 3-dimensional lattice conformation. Memantine is substituted with a methyl carbon at both R3 and R5; it contains an amine substitution at R1. Its name is derived from its structure, 3,5-dimethyladamantan-1-amine. Memantine is an arycyclohexylamine, belonging to the same category as ketamine and is a derivative of amantadine (adamantan-1-amine). It was originally synthesized in the late 1960s and like amantadine it is an adamantan-amine based uncompetitive NMDAR antagonists, used in the treatment of Alzheimer’s disease and other dementias and is considered to be ‘well-tolerated’.
79	Memantine extended release	memantine ER; Namenda XR	3,5-Dimethyladamantan-1-amine	Y	Y	N	See above
80	Menthol	Knaster Fresh	5-methyl-2-propan-2-ylcyclohexan-1-ol	N	N	Y	The menthol classic. Knaster Fresh is a minty blend of hemp aroma and Mentha Spicata. Arctic fresh.
81	Methylmorphenate		N/A	Y	Y	N	Methylmorphenate is a benzylmorpholine and is a derivative of the internationally controlled substance methylphenidate (methyl alpha-phenyl-2-piperidineacetate), where the piperidine ring has been replaced by a morpholine ring. Methylphenidate is used to treat attention-deficit hyperactivity disorder (ADHD).Methylmorphenate possesses two chiral carbons (stereocenters).
82	Methylnaphthidate	CHEMBL1183494; hdmp-28; DL-Threo-methylnaphthidate; Methyl 2-(naphthalen-2-yl)-2-(piperidin-2-yl)acetate; 2-Piperidineacetic acid, alpha-2-naphthalenyl-, methyl ester; BDBM50327107; ZINC29484313; and many others.	Methyl (naphthalen-2-yl)(piperidin-2-yl)acetate	Y	Y	N	HDMP-28 (methylnaphthidate) is structurally related to methylphenidate (controlled under the 1971 UN Convention) having a naphthalene ring instead of a benzene ring.HDMP-28 has been found to have reinforcing effects in a study that examined the reinforcing efficacy of psychostimulants in primate brain tissue.
83	Methylphenidate	Methylphenidan; Phenidylate; Concerta; Calocain; Daytrana; Plimasine; Meridil; Ritalin; Methyl phenidylacetate; Methylfenidan; Metilfenidato; Methylin; 113-45-1; Centedrin; Methylofenidan; Centedein; Tsentedrin; 4311/B Ciba; Metadate; Methylphen; Riphenidate; Centredin; Methyl alpha-phenyl-alpha-(2-piperidyl)acetate; alpha-Phenyl-2-piperidineacetic acid methyl ester; NCI-C56280; 2-Piperidineacetic acid, alpha-phenyl-, methyl ester; HSDB 3126; EINECS 204-028-6; C 4311; CHEMBL7	Methyl phenyl(piperidin-2-yl)acetate	N	N	Y	Methylphenidate, sold under various trade names, Ritalin being one of the most commonly known, is a central nervous system (CNS) stimulant of the phenethylamine[3] and piperidine classes that is used in the treatment of attention deficit hyperactivity disorder (ADHD) and narcolepsy. The original patent was owned by CIBA, now Novartis Corporation. It was first licensed by the US Food and Drug Administration (FDA) in 1955 for treating what was then known as hyperactivity. Methylphenidate’s mechanism of action involves the inhibition of catecholamine reuptake, primarily as a dopamine reuptake inhibitor. Methylphenidate acts by blocking the dopamine transporter and norepinephrine transporter, leading to increased concentrations of dopamine and norepinephrine within the synaptic cleft. This effect in turn leads to increased neurotransmission of dopamine and norepinephrine.[10] Methylphenidate is also a weak 5HT1A receptor agonist.
84	Mexamine	meksamin; 5-Methoxytryptamine; 5-MT; 2-(5-Methoxy-1H-indol-3-yl)ethanamine; meksamin; 5-Methoxytryptamine; 5-MT; 2-(5-Methoxy-1H-indol-3-yl)ethanamine;	2-(5-Methoxy-1H-indol-3-yl)ethanamine	N	Y	N	5-Methoxytryptamine, a tryptamine derivative that naturally occurs in the body at low levels. Apparently enhances dreams. 5-Methoxytryptamine (5-MT), also known as mexamine, is a tryptamine derivative closely related to the neurotransmitters serotonin and melatonin.
85	Modafinil	2-benzhydrylsulfinylethanamide; Modiodial; Provigil; Alertec; CRL-40476; Diphenylmethylsulfinylacetamide	2-[(Diphenylmethyl)sulfinyl]acetamide	Y	N	N	Modafinil is a synthetic stimulant which is also known under the trade names Modiodial in Europe and Provigil in the United Kingdom. It is approved for use in the treatment of daytime sleepiness associated with narcolepsy. Research has also suggested that modafinil may be effective in the treatment of sleepiness disorders other than narcolepsy i.e. idiopathic hypersomnia, night-shift sleep disorder, obstructive sleep disorder, obstructive sleep apnoea, multiple sclerosis, Parkinson’s disease, myotonic dystrophy, depression, schizophrenia, attention-deficit disorder and cocaine dependence and withdrawal.
86	Modafinil Sulphone	CRL 41056 and USP Modafinil Related Compound B	2-[(diphenylmethyl)sulfonyl]acetamide	Y	Y	N	Modafinil sulphone is considered to be one of two metabolites of modafinil, it being the minor metabolite. Modafinil sulphone is structurally related to the previously notified modafiendz which is the bis-fluoro-N-methyl analogue of modafinil.The properties of modafinil sulphone have not been described extensively in the literature. It has been reported that this substance has anticonvulsant activity and therefore may find use in the “treatment of preclinical subconvulsive manifestations”, further studies are required in this area. In other research, it has been stated that modafinil sulphone is pharmacologically active with a half-life of approximately 12 hours but it has been reported that it may also not exert any significant activity in the brain or periphery.
87	Mucuna pruriens	Velvet beans; Cow Itch; Itching Bean; Nescafe; Bengal velvet bean; Florida velvet bean; Mauritius velvet bean; Yokohama velvet bean; cowage; lacuna bean; Lyon bean	N/A	N	N	Y	Mucuna pruriens is a tropical vine growing from 3-18 meters with white to dark purple hanging flowers. It’s bean-like pods are covered with long stinging hairs and contain black, white, or tan seeds. The leaves, seeds, stems and roots contain L-Dopa, Serotonin, 5-HTP, and Nicotine, as well as N,N-DMT, Bufotenin, and 5-MeO-DMT. It has a tradition of use as a Ayurvedic aphrodesiac, treatment for parkinsons, ayahuasca admixture, and coffee substitute.
88	**N-methyl-4,4’-difluoro-modafinil**	N-Methylbisfluoromodafinil; Dehydroxyfluorafinil; Modafiendz N-meethyl-4,4-difluoro-modafinil	2-{[bis(4-fluorophenyl)methyl]sulfinyl}-N-methylacetamide	Y	Y	N	2-{[bis(4-fluorophenyl)methyl]sulfinyl}-N-methylacetamide is the bis-fluoro-N-methyl analogue of the substance modafinil and is currently marketed by online sellers as a nootropic substance called ‘modafiendz’. Modafinil is used to treat excessive sleepiness caused by narcolepsy, shift work sleep disorder and obstructive sleep apnea/hypopnea and is marketed as a prescription medication under a number of names in the EU including; Modasomil, Modiodal, Modiwake, Provigil and Vigil.
89	**N-methyl-cyclazodone**	N-methyl-cyclazodone;2-(cyclopropyl(methyl)amino)-5-phenyloxazol-4(5H)-one	N-methyl-cyclazodone;2-(cyclopropyl(methyl)amino)-5-phenyloxazol-4(5H)-one	Y	Y	N	N-methyl-cyclazodone is an approximately 3x - 5x more potent derivative of the nootropic and psychostimulant compound Pemoline.
90	Naringin	Kanna 50X Tincture	(2S)-7-[(2S,3R,4S,5S,6R)-4,5-dihydroxy-6-(hydroxymethyl)-3-[(2S,3R,4R,5R,6S)-3,4,5-trihydroxy-6-methyloxan-2-yl]oxyoxan-2-yl]oxy-5-hydroxy-2-(4-hydroxyphenyl)-2,3-dihydrochromen-4-one	N	N	Y	A tincture is one of the most convenient ways to get your dose of Kanna. Kanna extract induces a general feeling of euphoria and sociability, relaxation and increased focus, coupled with an energetic feeling. All natural: No chemicals or sugars have been added. Ingredients: Sceletium tortuosum extract, distilled water, naringin, glycerin, alcohol (14%). Content: 10ml (2 doses)
91	**Nefiracetam**	DM-9384	N-(2,6-dimethylphenyl)-2-(2-oxopyrrolidin-1-yl)acetamide	N	N	Y	Nootropic compound of the racetam family. Seems to enhance both GABAergic and cholinergic signalling. Long term use appears to be neuroprotective. Fat soluble.
92	Nicotine	Nicotine; tabak; tabacco; cigarettes; tobacco; Mapacho	(S)-3-[1-Methylpyrrolidin-2-yl]pyridine	N	N	Y	Nicotine (3-[(2S)-1-methylpyrrolidin-2-yl]pyridine) is a naturally occurring bicyclic compound comprised of a pyridine ring attached to the second carbon of a pyrrolidine ring that has a methyl substituent on the nitrogen. Pyridine is an unsaturated six-membered ring structurally related to benzene but with a nitrogen member. Nicotine additionally contains a substituted pyrrolidine ring, which is a saturated five-membered ring with one nitrogen member. These rings are bridged from the R3 position of the pyridine ring to the R2 position of the pyrrolidine ring. Tobacco is an annual or bi-annual growing 1-3 meters tall with large sticky leaves that contain nicotine. Native to the Americas, tobacco has a long history of use as a shamanic inebriant and stimulant. It is extremely popular and well-known for its addictive potential.
93	**Noopept**	GVS-111; Noopept; Noopept; GVS-111; Omberacetam	N-Phenylacetyl-L-prolylglycine ethyl este	Y	N	N	Noopept, or N-phenylacetyl-L-prolylglycine ethyl ester, is a synthetic peptide. A peptide is a chain of simple amino acids linked by peptide bonds. Noopept contains a phenylacetyl subunit bound to a small peptide chain of proline and glycine. The proline amino acid is composed of a carboxylic acid group bound to a pyrrolidine ring at C2. The glycine amino acid is bound to proline with a peptide bond and contains an amino group bound to the free carbon of ethanoic acid. Noopept is structurally similar to the endogenous neuropeptide cycloprolylglycine, for which it is a prodrug. Noopept is a dipeptide conjugate of piracetam although it is not a racetam as it lacks a pyrrolidone cycle.
94	NSI-189		(4-Benzylpiperazin-1-yl)-[2-(3-methylbutylamino)pyridin-3-yl]methanone	Y	Y	N	NSI-189 is considered an experimental drug that is currently being investigated by Neuralstem Inc., for the treatment of major depressive disorders (MDD). The research into this drug has been funded by the Defense Advanced Research Projects Agency (DARPA) and the National Institutes of Health (NIH) in the United States. Neuralstem states that NSI-189 “enables the creation of neural stem cell lines from many areas of the CNS, including the hippocampus” and “boost the generation of new neurons”. This would be achieved by the stimulation of neurogenesis of human hippocampus-derived neural stem cells in-vitro and in-vivo. This drug by Neuralstem is the first to undergo clinical trials and the company plans to develop NS1-189 into an orally administrable drug for the treatment of MDD, and other cognitive disorders such as Alzheimer’s disease and PTSD.
95	Octopamine	b-HO-HPEA	4-(2-amino-1-hydroxyethyl)phenol	N	N	Y	Octopamine is an organic chemical closely related to norepinephrine. In many types of invertebrates it functions as an important neurotransmitter and hormone, but in the human body it normally exists only at trace levels and has no known function. Because it shares some of the actions of norepinephrine, octopamine has been sold under trade names such as Epirenor, Norden, and Norfen for use as a sympathomimetic drug, available by prescription. In mammals, octopamine may mobilize the release of fat from adipocytes (fat cells), which has led to its promotion on the internet as a slimming aid. However, the released fat is likely to be promptly taken up into other cells, and there is no evidence that octopamine facilitates weight loss. It is also used to treat hypotension and as a cardiotonic.
96	**Oxiracetam**	BCP06209; HY-B1715; Tox21_110218; Tox21_500933; AC-670; AN-929; CGP 21690; CO0043; CT 848; MFCD00242951; RW2749; s4270; AB05478; ACN-001375; CCG-205014; CS-8012; LP00933; MCULE-8223030209; RP17379; TRA0048419; KS-00000O50; 4- Hydroxy-2-oxopyrrolidine-N-acetamide; 4-hydroxypyrrolidin-2-on-1-yl acetamide and many others.	(RS)-2-(4-hydroxy-2-oxopyrrolidin-1-yl)acetamide	N	N	Y	Oxiracetam, or (RS)-2-(4-hydroxy-2-oxopyrrolidin-1-yl)acetamide, is a synthetic compound of the racetam family. Racetams share a pyrrolidine nucleus, a five member nitrogenous ring with a ketone bonded oxygen at R2. This 2-pyrrolidone ring is bound to the terminal carbon of an acetamide group, an ethyl amide chain with a ketone bond (C=O) at the alpha carbon. Oxiracetam is substituted with an additional hydroxy group at R4, which is a chiral center for the molecule. Oxiracetam is presumably produced as a racemate of its enantiomers. Oxiracetam is structurally analogous to piracetam, which lacks the R4 hydroxy substitution of oxiracetam.
97	Peganum harmala	Syrian Rue	N/A	N	Y	N	Syrian rue (Peganum harmala) is a desert plant that grows from the Eastern Mediterranean, throughout the Middle East and up to India, Mongolia and Manchuria. The seeds have a long history of ritual and medicinal use, mainly as an incense. The smoke is widely believed to ward off the evil eye. The brown, triangular seeds contain a high amount of harmala alkaloids that have a MAO inhibiting effect. For this reason Syrian rue became popular among western psychonauts as an ayahuasca analogue.
98	Pemoline	SCHEMBL41636; Pemoline (JAN/USAN/INN); MLS000759491; MLS001424026; Pemoline, >=98% (HPLC); DTXSID3023427; NRNCYVBFPDDJNE-UHFFFAOYSA-; HMS2051C08; HMS3393C08; AOB87716; NSC25159; 5-Phenyl-2-imino-4-oxo-oxazolidin; and many others.	(RS)-2-amino-5-phenyl-1,3-oxazol-4(5H)-one	N	N	Y	A stimulant of the 4-oxazolidinone class. Was used as a medication for ADHD and Narcolepsy, yet was pulled from most markets due to liver failures among children.
99	Periwinkle	Madagascar Periwinkle; Rosy Periwinkle	N/A	N	N	Y	Periwinkle is a very common creeping perennial with dark green leaves and white, pink, to purple flowers. It has a long history of use, although it is not commonly used as an herbal remedy in modern treatments. The plant contains alkaloids and tannins, with a major alkaloid being vincamine, related to the semisynthetic vinpocetine.
100	**Phenibut**	Fenibut, Phenybut, PhGABA; b-Phenyl-g-aminobutyric acid	4-Amino-3-phenylbutanoic acid	N	Y	N	Phenibut is a derivative of GABA with a phenyl group in the b-position. It is a chiral molecule and thus has two potential configurations, as (R)- and (S)-enantiomers. It has almost the same structure of baclofen (lacking only a chlorine atom in the para-position of the phenyl group) and contains phenethylamine in its structure.Pregabalin has the same structure as phenibut, except that the phenyl group is instead an isobutyl group.
101	Phenylalanine		(S)-2-Amino-3-phenylpropanoic acid	N	N	Y	Phenylalanine is found naturally in the breast milk of mammals. It is used in the manufacture of food and drink products and sold as a nutritional supplement for its reputed analgesic and antidepressant effects. It is a direct precursor to the neuromodulator phenethylamine, a commonly used dietary supplement. As an essential amino acid, phenylalanine is not synthesized *de novo* in humans and other animals, who must ingest phenylalanine or phenylalanine-containing proteins.
102	Phenylpiracetam	phenyl-; BRN 5030440; (2-Oxo-4-phenylpyrrolidin-1-yl)acetamide; 2-(2-Oxo-4-phenyl-pyrrolidin-1-yl)-acetamide; AK-81769; J-500892; Carphedone; Fonturacetam [INN]; phenypiracetam; Carphedo; ACMC-1BLAK; AC1Q4ZOM; AC1L30ZV; Oprea1_208829; Oprea1_429090; MLS000113218; and many others.	(R,S)-2-(2-oxo-4-phenylpyrrolidin-1-yl)acetamide	N	N	Y	Phenylpiracetam is based on the piracetam molecular skeleton with an additional phenyl group attached to the pyrrolidone nucleus, albeit at a different steric location than the substituted phenyl groups observed on aniracetam or nefiracetam. Due to the chiral center at the fourth position of the pyrrolidinone ring, it can exist in an S or R-isomer; the clinically used form is the racemic mixture.[6]
103	**Picamilon**	GABA-NG; N-(3-Carboxypropyl)nicotinamide; UNII-0S5N9SEK4N; 0S5N9SEK4N; 4-[(pyridin-3-ylcarbonyl)amino]butanoic acid; Butanoic acid, 4-[(3-pyridinylcarbonyl)amino]-; N-nicotinoyl-gamma-aminobutyric acid; 4-(pyridin-3-ylformamido)butanoic acid; 4-(pyridine-3-carbonylamino)butanoic acid; 4-[(Pyridine-3-carbonyl)amino]butyric acid; Butanoic acid, 4-((3-pyridinylcarbonyl)amino)-; and many others.	4-(Pyridine-3-carbonylamino)butanoic acid	N	N	Y	An analogue of GABA that does pass the brain blood barrier, which is then hydrolyzed into GABA and Niacin. In which the GABA could produce an anxiolytic effect. The Niacin as a vasodilator. And is usually used as part of a nootropic stac
104	**Piracetam**	MolPort-000-839-314; NINDS_000259; HMS1569L15; HMS1921L12; HMS2092D18; HMS2096L15; HMS2230B24; HMS3262N20; HMS3371G01; HMS3657A05; HMS3713L15; and many others.	2-(2-Oxopyrrolidin-1-yl)acetamide	N	N	Y	Piracetam, or 2-oxo-1-pyrrolidine-acetamide, is a synthetic compound of the racetam family. Racetams share a pyrrolidine nucleus, a five member nitrogenous ring with a ketone bonded oxygen at R2.[3] This 2-pyrrolidone ring is bound to the terminal carbon of an acetamide group, an ethyl amide chain with a ketone bond (C=O) at the alpha carbon.
105	**Pramiracetam**	68497-62-1; amacetam; Pramiracetam [INN]; Pramiracetamum [INN-Latin]; UNII-4449F8I3LE; 1-Pyrrolidineacetamide, N-(2-(bis(1-methylethyl)amino)ethyl)-2-oxo-; 4449F8I3LE; C14H27N3O2; Neupramir; Pramiracetam (INN); N-(2-(diisopropylamino)ethyl)-2-(2-oxopyrrolidin-1-yl)acetamide; Pramiracetam hydrate; N-[2-[di(propan-2-yl)amino]ethyl]-2-(2-oxopyrrolidin-1-yl)acetamide; and many others	N-[2-(Diisopropylamino)ethyl]-2-(2-oxopyrrolidin-1-yl)acetamide	N	N	Y	Pramiracetam, or N-[2-(Diisopropylamino)ethyl]-2-(2-oxopyrrolidin-1-yl)acetamide, is a synthetic compound of the racetam family. Racetams share a pyrrolidine nucleus, a five member nitrogenous ring with a ketone bonded oxygen at R2. This 2-pyrrolidone ring is bound to the terminal carbon of an acetamide group, an ethyl amide chain with a ketone bond (C=O) at the alpha carbon. Pramiracetam features an additional substituion bonded to RN of the acetamide group of a ethyl amide chain with two isopropyl carbon chains attached to the terminal nitrogen. Pramiracetam is structurally analogous to piracetam with an added diisopropyl ethylamino chain.Pramiracetam is prepared from piracetam by substituting the amide group with a dipropan-2-ylaminoethyl group.
106	PRE-084	PRE-084 Hydrochloride; Pre-084; PRE-084 (hydrochloride); 138847-85-5; Pre 084; 75136-54-8; 2-(4-Morpholinethyl) 1-phenylcyclohexanecarboxylate hydrochloride; SR-01000076063; PRE-084, solid; MLS000860067; SCHEMBL7381926; CHEMBL1449159; CTK8E9795; MolPort-003-959-092; BCP16863; Tox21_500927; HY-18100A; and many others	2-morpholin-4-ylethyl 1-phenylcyclohexane-1-carboxylate	Y	N	N	A sigma-1 receptor agonist derived structurally from PCP. It has cognitive enhancing effects as well as antidepressant effects, and shows promise in treating many nervous system diseases such as ALS and parkinsons.
107	**PRL-8-53**	Prl-8-53; 51352-88-6; methyl 3-[2-[benzyl(methyl)amino]ethyl]benzoate; methyl 3-{2-[benzyl(methyl)amino]ethyl}benzoate; AC1L22UU; AC1Q5Z4V; 9043AF; ZINC31982738; AJ-32456; 3-[2-[Benzyl(methyl)amino]ethyl]benzoic acid methyl ester	Methyl 3-[2-[benzyl(methyl)amino]ethyl]benzoate	N	N	Y	A nootropic research chemical first synthesized in the 70s. One study shows a drastic improvement in mid-term memory among users, but otherwise it is severely lacking in information surrounding it. It has no recreational potential.
108	**Prolintane**	Prolintane; Prolintane; Phenylpyrrolidinopentane; Catovit; Katovit; Promotil; Villescon	1-Phenyl-2-pyrrolidinylpentane	N	N	Y	1-Phenyl-2-pyrrolidinylpentane (also known as Prolintane or Pyrrolidinopentiophenone, and by the trade names Catovit, Promotil, and Villescon) is a synthetic central nervous system (CNS) stimulant that is structurally similar to the substituted pyrrolidine class of compounds such as MDPV and A-PVP albeit with notably attenuated effects. Prolintane was first synthesized in the 1950s, where it was found primarily to act as as a norepinephrine-dopamine reuptake inhibitor (NDRI)[1] which is thought to confer it stimulant and potential nootropic qualities.
109	Propylphenidate	PPH	N/A	Y	Y	N	Propylphenidate is structurally related to isopropylphenidate from the piperidine and pyrrolidine category of new psychoactive substances. Propylphenidate and isopropylphenidate are also structurally related to methyl- and ethylphenidate, where the isopropyl or propyl is replaced with a methyl or ethyl group. Propylphenidate can be synthesised from methylphenidate.
110	**PWZ-029**	CHEMBL45346; 6H-Imidazo[1,5-a][1,4]benzodiazepin-6-one, 8-chloro-4,5-dihydro-3-(methoxymethyl)-5-methyl-; SCHEMBL6847260; BDBM50034820; 3-Methoxymethyl-5-methyl-8-chloro-4,5-dihydro-6H-imidazo[1,5-a][1,4]benzodiazepin-6-one	8-Chloro-3-(methoxymethyl)-5-methyl-4,5-dihydro-6H-imidazo[1,5-a][1,4]benzodiazepin-6-one	N	N	Y	PWZ-029 is a benzodiazepine derivative drug with nootropic effects developed by WiSys,[1] It acts as a subtype-selective, mixed agonist-inverse agonist at the benzodiazepine binding site on the GABAA receptor, acting as a partial inverse agonist at the α5 subtype and a weak partial agonist at the α3 subtype. This gives it a mixed pharmacological profile, producing at low doses memory-enhancing effects but with no convulsant or anxiogenic effects or muscle weakness, although at higher doses it produces some sedative effects.
111	Quercetin	Trichocereus Bridgesii Cutting; Trichocereus Bridgesii Monstruosus Cutting	2-(3,4-dihydroxyphenyl)-3,5,7-trihydroxychromen-4-one	N	N	Y	Description Endemic to the high Andean region of La Paz, the Bolivian torch is a lesser known psychedelic cactus containing several alkaloids including the psychoactive alkaloid mescaline. Bioassay reports indicate that Trichocereus bridgesii is much more potent than the measured mescaline content suggests. Capable of producing a mind-expanding kaleidoscopic psychedelic experience, the effects of the Bolivian torch have been described as more clear-headed than the Peruvian torch. In Bolivia, the cactus is used as a hedge plant, serving a practical and decorative function.
112	Quetiapine	Xeroquel; 2-{2-[4-(Dibenzo[b;f][1;4]thiazepin-11-yl)-1-piperazinyl]ethoxy}ethanol; 11-[4-[2-(2-hydroxyethoxy)ethyl]-1-piperazinyl]dibenzo[b;f][1;4]thiazepine; ICI 204;636 Ketipinor; quetiapine; quetiapinum; ZM 204;636; Seroquel	2-[2-(4-benzo[b][1,4]benzothiazepin-6-ylpiperazin-1-yl)ethoxy]ethanol	Y	N	N	Quetiapine is a dibenzothiazepine derivative. It is an atypical antipsychotic agent and medicinal products containing quetapine as the active pharmaceutical ingredient (as quetiapine fumarate or quetiapine hemifumarate) have been authorised in a number of Member States for the treatment of schizophrenia and for the prevention and treatment of bipolar disorder.
113	R-alpha-lipoic acid	Core Memory Nootropics	5-[(3R)-dithiolan-3-yl]pentanoic acid	N	N	Y	BrainBullets Core are capsules specially designed for anyone who appreciates the importance of a healthy brain. Core optimises your memory and protects your brain. In this way you will give your upper deck the essential vitamins it needs every day to keep your focus and concentration at peak level. BrainBullets Core can be ordered in packs of 15 or 30. Take one capsule each day to maintain your cognitive performance in peak condition.
114	**Razobazam**	UNII-LZ84VWN0U4; Hoe 175; 78466-98-5; LZ84VWN0U4; 4,8-Dihydro-3,8-dimethyl-4-phenylpyrazolo(3,4-b)(1,4)diazepine-5,7(1H,6H)-dione; Pyrazolo(3,4-b)(1,4)diazepine-5,7(1H,6H)-dione, 4,8-dihydro-3,8-dimethyl-4-phenyl-; 5662; and many others.	C14H14N4O2 3,8-Dimethyl-4-phenyl-2,8-dihydropyrazolo[3,4-b][1,4]diazepine-5,7(4H,6H)-dione	N	N	Y	It is a drug which is a benzodiazepine derivative. Its mechanism of action appears to be quite different from that of most benzodiazepine drugs, and it produces nootropic effects in animal studies.
115	Rimonabant	SR-141716A; Acomplia; SR141,716; Zimulti	5-(4-Chlorophenyl)-1-(2,4-dichloro-phenyl)-4-methyl-N-(piperidin-1-yl)-1H-pyrazole-3-carboxamide	Y	Y	N	Rimonabant is the active pharmaceutical ingredient in the medicinal product Acomplia, which was suspended from the market in the European Union in November 2008 over concerns of serious psychiatric disorders (including depression, sleep disturbances, anxiety, and aggression) associated with its use. Acomplia was withdrawn from the market in January 2009.
116	RO-4491533	2H-1,5-Benzodiazepin-2-one, 4-[3-(2,6-dimethyl-4-pyridinyl)phenyl]-1,3-dihydro-7-methyl-8-(trifluoromethyl)-; RO4491533; CHEMBL1629855; D09YEQ; GTPL6226; SCHEMBL5562484; BDBM50332963; LYTVXCQQTLUEQR-UHFFFAOYSA-N	4-[3-(2,6-Dimethylpyridin-4-yl)phenyl]-7-methyl-8-(trifluoromethyl)-1,3-dihydro-2H-1,5-benzodiazepin-2-one	N	N	Y	It is a drug developed by Hoffmann-La Roche which acts as a potent and selective negative allosteric modulator for group II of the metabotropic glutamate receptors (mGluR2/3), being equipotent at mGluR2 and mGluR3 but without activity at other mGluR subtypes. In animal studies, RO-4491533 produced antidepressant effects and reversed the effects of the mGluR2/3 agonist LY-379,268 with similar efficacy but slightly lower potency than the mGluR2/3 antagonist LY-341,495.
117	**RO4938581**	9H-Imidazo[1,5-a][1,2,4]triazolo[1,5-d][1,4]benzodiazepine, 3-bromo-10-(difluoromethyl)-; CHEMBL1080588; 883093-10-5; RO 4938581; D0E7PY; 4-tert.Butylcalix[8]arene; GTPL4299; SCHEMBL2426998; EX-A856; AFJRYPJIKHMNGL-UHFFFAOYSA-N; BDBM50311045; and many others.	3-Bromo-10-(difluoromethyl)-9H-imidazo[1,5-a][1,2,4]triazolo[1,5-d][1,4]benzodiazepine	N	N	Y	Ro4938581 is a nootropic drug invented in 2009 by a team working for Hoffmann-La Roche, which acts as a subtype-selective inverse agonist at the α5 subtype of the benzodiazepine binding site on the GABAA receptor. It has good selectivity for the α5 subtype and did not produce convulsant or anxiogenic effects in animal studies, making it a promising potential nootropic.[1][2][3] Ro4938581 and a related derivative basmisanil (RG-1662, RO5186582) have subsequently been investigated for the alleviation of cognitive dysfunction in Down syndrome
118	**S-Adenosyl methionine**	SAM-e, Methylguanidoacetic acid; S-Adenosyl methionine; SAM-e; Methylguanidoacetic acid	(2S)-2-Amino-4-(((2S,3S,4R,5R)-5-(6-aminopurin-9-yl)-3,4-dihydroxyoxolan-2-yl)methyl-methylsulfonio)butanoate	N	N	Y	S-adenosyl methionine is a molecule, found endogenously as a substrate synthesized by the sub-groups adenosine and methionine through an the enzyme methionine adenosyltransferase. The adenosine subcomponent is comprised of an adedine nucleobase bonded to a ribose chain. This ribose chain is attached to the terminal carbon of the methionine group. Methionine is a butyl carboxylic acid substituted at R2 with an amino group and at R4 with a methylthio (carbon-sulphur) group. S-adenosyl methionine is an essential methyl donator in metabolic reactions.
119	Sakae naa	Combretum quadrangulare	3-benzyl-1-hydroxy-2H-pyridine-4-carbaldehyde	N	N	Y	Sakae Naa (Combretum quadrangulare) is a small tree native to Southeast Asia, the leaves of which are reportedly used as a substitute for kratom in areas where kratom is banned. There is dispute about whether its effects are similar to the effects of kratom.
120	Selegiline	Deprenyl; Eldepryl; Emsam; L-deprenyl	(R)-N,a-dimethyl-N-2-propynylbenzeneethanamine	N	N	Y	Selegiline increases the actions of dopamine in the brain by inhibiting the enzymes that break it down. After being sold for years as an anti-aging supplement and a treatment for Parkinson’s disease, the FDA approved use of a Selegiline transdermal patch (Emsam) for treatment of depression in 2006.
121	**SH-053-R-CH3-2′F**	4H-Imidazo[1,5-a][1,4]benzodiazepine-3-carboxylic acid, 8-ethynyl-6-(2-fluorophenyl)-4-methyl-, ethyl ester, (4R)-; 872874-14-1; SCHEMBL7718347; NGYKELBMVXBFSM-CQSZACIVSA-N; ZINC35847341; KB-275294	Ethyl 8-ethynyl-6-(2-fluorophenyl)-4-methyl-4H-imidazo[1,5-a][1,4]benzodiazepine-3-carboxylate	N	N	Y	SH-053-R-CH3-2′F is a drug used in scientific research which is a benzodiazepine derivative. It produces some of the same effects as other benzodiazepines, but is much more subtype-selective than most other drugs of this class, having high selectivity, binding affinity and efficacy at the α5 subtype of the GABAA receptor. This gives much tighter control of the effects produced, and so while SH-053-R-CH3-2′F retains sedative and anxiolytic effects, it does not cause ataxia at moderate doses.[1] SH-053-R-CH3-2′F also blocks the nootropic effects of the α5-selective inverse agonist PWZ-029, so amnesia is also a likely side effect.
122	Sildenafil	Viagra; Aphrodil	1-[[3-(6,7-dihydro-1-methyl-7-oxo-3-propyl-1H-pyrazolo [4,3-d]pyrimidin- 5-yl)-4-ethoxyphenyl]sulfonyl]-4- methylpiperazine citrate	N	N	Y	Sildenafil citrate is prescribed in the treatment of Erectile Dysfunction. It is commonly referred to as a “lifestyle drug”. Lifestyle drugs are prescribed for quality-of-life conditions such as baldness, impotence, obesity and smoking cessation. Sildenafil is a synthetic piperazine derivative.
123	St John’s wort	St John’s Wort; Sint Janskruid	N/A	N	N	Y	St. John’s wort, also known as “perforate” or “common” St. John’s wort, is a plant with yellow flowers, that some people, at first glance, may mistake for a common, roadside weed. It has been used for centuries as an herbal mood enhancer. Enjoy the rich flavour and effects of one of Mother Nature’s miracle plants.
124	Stablon	Tianeptine, Stablon, Coaxil, Tatinol; Tianeptine; Stablon; Coaxil; Tatinol;Tianeptine	(RS)-7-(3-chloro-6-methyl-6,11-dihydrodibenzo[c,f][1,2]thiazepin-11-ylamino)heptanoic acid S,S-dioxide	Y	N	N	In terms of molecular structure and chemistry, tianeptine is a tricyclic antidepressant as its molecular structure is composed of three cyclic compounds. Despite tianeptine’s chemical similarity to other TCAs, its effects and mechanisms are fairly unique.
125	**Sulbutiamine**	sulbut; arcalion; enerion; bisibuthiamine; youvitan	4-[(4-amino-2-methyl-pyrimidin-5-yl)methyl-formyl-amino]-3-[2-[(4-amino-2-methyl-pyrimidin-5-yl)methyl-formyl-amino]-5-(2-methylpropanoyloxy)pent-2-en-3-yl]disulfanyl-pent-3-enyl] 2-methylpropanoate	N	N	Y	A thiamine derivative nootropic and stimulant drug. Caution should be used as Sulbutiamine reduces dopamine output over time with consistant usage.
126	Tadalafil	Cialis; Adcirca; Tadacip	(6R,12aR)-6-(1,3-benzodioxol-5-yl)-2-methyl-2,3,6,7,12,12a-hexahydropyrazino[1’,2’:1,6] pyrido[3,4-b]indole-1,4-dione	N	N	Y	Tadalafil, a PDE5 inhibitor used to combat erectile disfunction. Dangerous in combination with other drugs which lower blood pressure.
127	Tannic acid	Guarana Powder [Paulinnia Cupana]	[2,3-dihydroxy-5-[[(2R,3R,4S,5R,6S)-3,4,5,6-tetrakis[[3,4-dihydroxy-5-(3,4,5-trihydroxybenzoyl)oxybenzoyl]oxy]oxan-2-yl]methoxycarbonyl]phenyl] 3,4,5-trihydroxybenzoate	N	N	Y	Use Guarana to get more energy. People who like a boost can use this! It is now available in powder form. Dissolve it in hot water, tea, hot milk, coffee or in any another type of drink. Best is to first take one drink. Then you can experience the effect on your body. If the effect is pleasant, you can always try another drink.
128	**Theanine**	Theanine, L-Theanine, L-g-glutamylethylamide and N5-ethyl-L-glutamine; Theanine; L-Theanine; L-g-glutamylethylamide and N5-ethyl-L-glutamine	N-ethyl-L-glutamine; (2S)-2-ammonio-5-(ethylamino)-5-oxopentanoate	N	N	Y	Theanine, or N-ethyl-L-glutamine, is an amino acid analogue of L-glutamine. Its structure is comprised of a five carbon straight chain carboxylic acid called pentanoic acid, which is bonded to an amino group at R2, and an additional ketone group at R5. Also substituted at R5 of the pentanoic group is an ethylamino chain connected at its amino constituent. Theanine is understood to refer to the levorotary enantiomer, which is well documented, rather than the relatively unresearched dextrorotary enantiomer.
129	**Tolibut**	28311-38-8; 4-amino-3-(4-methylphenyl)butanoic acid; 4-amino-3-(p-tolyl)butanoic acid; AC1L2LNB; Oprea1_567181; SCHEMBL18386267; 28311-37-7 (hydrochloride); and many others	4-Amino-3-(4-methylphenyl)butanoic acid	N	N	Y	Analogue of GABA, and the 4-methyl analogue of Phenibut. Has similar effects, acts on GABA(B).
130	Tropicamide	N-Ethyl-alpha-(hydroxymethyl)-N-(4-pyridinylmethyl) benzeneacetamide; Visumidriatic; Mydriaticum; Mydriafair; Tropicacyl; Mydriacyl; Paremyd; Minims tropicamide; Mydrum; Bistropamide	(RS)-N-ethyl-3-hydroxy-2-phenyl-N-(pyridin-4-ylmethyl)propenamide	Y	N	N	Medicinal products containing tropicamide are authorised in the European Union. It is an antimuscarinic used in medicinal products to dilate the pupils, specifically as a topical mydriatic and cycloplegic.
131	Tryptophan	2-Amino-3-(1H-indol-3-yl)propanoic acid; Trp; W; TRP	(2S)-2-amino-3-(1H-indol-3-yl)propanoic acid	N	N	Y	Tryptophan (symbol Trp or W)[2] is an α-amino acid that is used in the biosynthesis of proteins. Tryptophan contains an α-amino group, an α-carboxylic acid group, and a side chain indole, making it a non-polar aromatic amino acid. It is essential in humans, meaning the body cannot synthesize it: it must be obtained from the diet. Tryptophan is also a precursor to the neurotransmitter serotonin, the hormone melatonin and vitamin B3.[3] It is encoded by the codon UGG.
132	**Tyrosine**	L-Tyrosine or 4-hydroxyphenylalanine; Tyrosine; L-Tyrosine or 4-hydroxyphenylalanine	L-Tyrosine	N	N	Y	Tyrosine is a non-essential phenylalanine-derived amino acid. Tyrosine’s structure is made a para-hydroxylated phenyl ring connected to a pentanoic acid group, which is a five member carbon chain with a carboxyl (C(=O)OH) group on the terminal carbon. This pentanoic acid chain is substituted at R2 with an amino group in levorotary orientation.
133	**URB-597**	KDS-4103; Cyclohexylcarbamic acid 3’-carbamoyl-biphenyl-3-yl ester	[3-(3-Carbamoylphenyl)phenyl] N-cyclohexylcarbamate	Y	N	N	URB-597 belongs to the carbamate chemical class. It is known to be an inhibitor of the enzyme FAAH (fatty-acid amide hydrolase) and this includes the metabolic hydrolysis of the endocannabinoid/anandamide/(a fatty-acid amide). It has been used extensively in neuropharmacological research into endocannabinoid system. It’s also known as KDS-4103. KDS-4103 is being developed by Kadmus Pharmaceuticals, Inc. for clinical trials in humans.
134	Vardenafil	Valif 20	2-[2-ethoxy-5-(4-ethylpiperazin-1-yl)sulfonylphenyl]-5-methyl-7-propyl-3H-imidazo[5,1-f][1,2,4]triazin-4-one	N	N	Y	Besides Cialis (Tadalafil) we now offer Valif 20 (Vardenafil). Valif 20 improves erection and results in successful intercourse. Valif 20 is also known as Vardenafil. The effects of Valif 20 are noticeable 15 to 25 minutes after ingestion. In the absence of sexual arousal Valif 20 will not work optimally. So make sure you are in a horny environment
135	Vasopressin	ADH; Pressyn; Diapid	(2S)-2-[(4R)-2-oxo-4-propylpyrrolidin-1-yl]butanamide	N	N	Y	Vasopressin is a hormone produced in the hypothalamus which increases water retention. asopressin is used to manage anti-diuretic hormone deficiency. It has off-label uses and is used in the treatment of gastrointestinal bleeding, ventricular tachycardia and ventricular defibrillation.
136	Vinpocetine	Cavinton; ethyl apovincaminate	ethyl (15S,19S)-15-ethyl-1,11-diazapentacyclo[9.6.2.02,7.08,18.015,19]nonadeca-2,4,6,8(18),16-pentaene-17-carboxylate	N	N	Y	Vinpocetine is a derivative of vincamine from the periwinkle plant. It increases cerebral blood flow and is said to improve memory.
137	Vitamin A		(2E,4E,6E,8E)-3,7-dimethyl-9-(2,6,6-trimethylcyclohex-1-en-1-yl)nona-2,4,6,8-tetraen-1-ol	N	N	Y	Vitamins are organic (carbon-containing) molecules that are necessary for the functioning of the human body. They have a wide range of effects and functions.
138	Vitamin B12		alpha-(5,6-Dimethylbenzimidazolyl)cobamidcyanide	N	N	Y	See above
139	Vitamin D		(1S,3Z)-3-[(2E)-2-[(1R,3aS,7aR)-7a-methyl-1-[(2R)-6-methylheptan-2-yl]-2,3,3a,5,6,7-hexahydro-1H-inden-4-ylidene]ethylidene]-4-methylidenecyclohexan-1-ol	N	N	Y	See above
140	Withania somnifera	Ashwagandha, an Indian ginseng	(2R,6S,7R,9R,11S,15R,16S)-6-hydroxy-15-[(1S)-1-[(2R)-5-(hydroxymethyl)-4-methyl-6-oxo-2,3-dihydropyran-2-yl]ethyl]-2,16-dimethyl-8-oxapentacyclo[9.7.0.02,7.07,9.012,16]octadec-4-en-3-one	N	N	Y	Is an Adaptogen. It is commonly used for its ability to prevent anxiety. It also is helpful in relieving insomnia. It’s name means “Smell of Horse” due to its smell and the traditional belief that ingesting the this herb will give you the strength and virility of a horse.
141	Yerba mate	South American holly; Beverage: yerba mate; Paraguay tea; Braziliain tea; jesuit tea; ka’a (Guarani)	N/A	N	N	Y	Ilex paraguariensis is a small S. American tree that’s leaves contain caffeine and other xanthines. The toasted leaves have a long history of use as a stimulant tea. Falsely rumored to contain a unique chemical mateine.
142	Yohimbine HCL	Yohimbe	methyl (1S,15R,18S,19R,20S)-18-hydroxy-1,3,11,12,14,15,16,17,18,19,20,21-dodecahydroyohimban-19-carboxylate;hydrochloride	N	N	Y	Pausinystalia yohimbe is a West African tree thats bark contains yohimbine. It has a long history of human use as a stimulant and aphrodisiac. It is commonly sold as an herbal supplement to improve erectile function.

The 35 CEs described as nootropic in NPSfinder^®^ are in bold.
